# Low Red Blood Cell Vitamin C Concentrations Induce Red Blood Cell Fragility: A Link to Diabetes Via Glucose, Glucose Transporters, and Dehydroascorbic Acid

**DOI:** 10.1016/j.ebiom.2015.09.049

**Published:** 2015-10-03

**Authors:** Hongbin Tu, Hongyan Li, Yu Wang, Mahtab Niyyati, Yaohui Wang, Jonathan Leshin, Mark Levine

**Affiliations:** Molecular and Clinical Nutrition Section, Digestive Diseases Branch, Intramural Research Program, National Institute of Diabetes and Digestive and Kidney Diseases, National Institutes of Health (NIDDK, NIH)

**Keywords:** AA, ascorbic acid, DHA, dehydroascorbic acid, *GLUT*, facilitated glucose transporter, *Gulo*^*-*/*-*^, gulonolactone oxidase knockout mouse unable to synthesize ascorbate, PBS, phosphate buffered saline, RBCs, red blood cells, RIPA, Western blot cell lysis buffer, *SVCT*, sodium-dependent vitamin C transporter, TCEP, Tris(2-carboxyethyl)phosphine, 3-O-MG, 3-O-methylglucose, WT, wildtype mouse, Ascorbic Acid, Dehydroascorbic Acid, Red Blood Cells, Diabetes, Glucose Transport, β-Spectrin

## Abstract

Strategies to prevent diabetic microvascular angiopathy focus on the vascular endothelium. Because red blood cells (RBCs) are less deformable in diabetes, we explored an original concept linking decreased RBC deformability to RBC ascorbate and hyperglycemia. We characterized ascorbate concentrations from human and mouse RBCs and plasma, and showed an inverse relationship between RBC ascorbate concentrations and deformability, measured by osmotic fragility. RBCs from ascorbate deficient mice were osmotically sensitive, appeared as spherocytes, and had decreased β-spectrin. These aberrancies reversed with ascorbate repletion in vivo. Under physiologic conditions, only ascorbate's oxidation product dehydroascorbic acid (DHA), a substrate for facilitated glucose transporters, was transported into mouse and human RBCs, with immediate intracellular reduction to ascorbate. *In vitro*, glucose inhibited entry of physiologic concentrations of dehydroascorbic acid into mouse and human RBCs. *In vivo*, plasma glucose concentrations in normal and diabetic mice and humans were inversely related to respective RBC ascorbate concentrations, as was osmotic fragility. Human RBC β-spectrin declined as diabetes worsened. Taken together, hyperglycemia in diabetes produced lower RBC ascorbate with increased RBC rigidity, a candidate to drive microvascular angiopathy. Because glucose transporter expression, DHA transport, and its inhibition by glucose differed for mouse versus human RBCs, human experimentation is indicated.

## 1 Introduction

Diabetes type 2 is an epidemic public health problem. Poorly controlled diabetes results in accelerated microvascular disease and chronic debilitating morbidities and mortality (Beckman et al., 2002). Diabetic microvascular angiopathy is the leading cause of blindness, end stage renal disease and amputations worldwide, as well as myocardial infarction, stroke and peripheral arterial disease.

Preventing or delaying microvascular disease could improve the lives of millions, prevent catastrophic illness, and save billions of dollars. The pathogenesis of microangiopathy in diabetes is unknown. Clinical efforts are based on glycemic control. The research focus of some prevention efforts is the endothelium and its role in protecting blood vessels (Fioretto et al., 2010, Wong et al., 2010). Vascular smooth muscle abnormalities, platelet dysfunction, abnormal coagulation and impaired vascular repair are other pathologies proposed to lead to diabetic vasculopathy (Beckman et al., 2002, Cubbon et al., 2013). Oxidants generated by hyperglycemia within endothelial cells (Nishikawa et al., 2000) or other vascular cells may initiate endothelial cell damage (Yu et al., 2006, Wang et al., 2012).

Endothelial cell damage secondary to hyperglycemia can be one initiator of diabetic microangiopathy by impairing oxygen delivery, resulting in microvascular hypoxia. Another plausible pathway is that oxidants generated by hyperglycemia, from endothelial cells or others, impair oxygen delivery by affecting the delivery system itself: red blood cells (RBCs). In fact, substantial evidence indicates that diabetes induces changes in RBC structure and function through a progressive decline in RBC deformability (Peterson et al., 1977, McMillan et al., 1978, Kamada et al., 1992, Virtue et al., 2004, Diamantopoulos et al., 2004, Brown et al., 2005, Shin et al., 2007, Kung et al., 2009, Keymel et al., 2011, Buys et al., 2013). RBC deformability is vital to RBC function, and plays a major role in microvascular flow. Impaired deformability adversely affects capillary perfusion (Simchon et al., 1987, Parthasarathi and Lipowsky, 1999). Consistent with decreased deformability, diabetes is associated with increased RBC fragility and decreased RBC survival (Peterson et al., 1977, Parthasarathi and Lipowsky, 1999, Virtue et al., 2004, Kung et al., 2009). Because stiffer RBCs may compromise the microcirculation and oxygen delivery, strategies to improve RBC deformability could modify microvascular hypoxia, with direct clinical implications.

In this regard, there are underappreciated links between RBCs, vitamin C, and diabetes. Multiple reports describe lower vitamin C concentrations in diabetic subjects, especially those with microvascular complications such as retinopathy and nephropathy (Som et al., 1981, Ali and Chakraborty, 1989, Sinclair et al., 1991, Will and Byers, 1996, Lindsay et al., 1998, Cunningham, 1998, Chen et al., 2006). However, many datasets utilized unreliable vitamin C assays, making it difficult to interpret findings (Will and Byers, 1996, Padayatty et al., 2003). Diabetic vascular disease and vitamin C deficiency were tied together in an early hypothesis (Mann and Newton, 1975), but it lacked mechanism and supportive evidence. Consistent with a hypothesized role for a RBC deformability defect in diabetes, anemia and hemolysis are manifestations of vitamin C deficiency in humans, and in mice (*Gulo*^*−*/*−*^) unable to synthesize the vitamin (Chazan and Mistilis, 1963, Hart et al., 1964, Cox, 1968, Maeda et al., 2000). Unfortunately, deformability measures were not described in vitamin C deficient patients, and their clinical data are confounded by co-existent vitamin deficiencies.

Here we couple original links between diabetes and vitamin C with RBCs as a key cell type; oxidized vitamin C (dehydroascorbic acid, DHA) as a key transported substrate; and the chemical structure similarity between DHA and glucose (Vera et al., 1993, Rumsey et al., 1997, Corpe et al., 2013). For nearly all tissues, ascorbate transport is mediated by sodium-dependent vitamin C transporter *SVCT2* (Sotiriou et al., 2002). However, *SVCT2* is absent from RBCs (May et al., 2007). Because RBCs contain ascorbate (Li et al., 2012), another transport mechanism exists. It is likely that the product of ascorbate oxidation, dehydroascorbic acid, is transported on facilitated glucose transporters (*GLUTs*) and immediately reduced to ascorbate within RBCs (Hughes and Maton, 1968, Bianchi and Rose, 1986, Mendiratta et al., 1998). Based on expressed transporter data, hyperglycemia from diabetes could inhibit dehydroascorbic acid entry into RBCs (Vera et al., 1993, Rumsey et al., 1997). Some data do not support this rationale, but experiments were performed using DHA concentrations 2–3 orders of magnitude above physiological concentrations, and indirect assays that did not account for substrate degradation (Montel-Hagen et al., 2008a, Sage and Carruthers, 2014). Lower DHA concentrations could not be investigated due to assay limitations, also precluding accurate RBC ascorbate measurements (May et al., 2001, Montel-Hagen et al., 2008a, Li et al., 2012).

Utilizing a recently developed ultrasensitive assay for vitamin C in RBCs and physiologic transport conditions (Li et al., 2012), we propose an original multicomponent hypothesis linking vitamin C to diabetes. The parts of the hypothesis investigated here are whether low ascorbate concentrations occur in RBCs in comparison with other cells; whether low ascorbate concentrations in RBCs have a consequence; and whether and how low ascorbate RBC concentrations can be coupled to hyperglycemia in vitro and in vivo.

## 2 Methods

### 2.1 Materials

Ascorbic acid was purchased from Sigma/Aldrich. Dehydroascorbic acid was synthesized *de novo* from ascorbate immediately before each experiment (Li et al., 2012, Corpe et al., 2013). Antibodies were obtained from Abcam (Cambridge MA), Santa Cruz Biotechnology (Dallas TX), and Novus Biologicals (Littleton, CO). Antibodies from Abcam: anti-GLUT 1 antibody (ab652), anti-GLUT 2 antibody(ab54460), anti-GLUT 3 antibody (ab41525), anti-GLUT 4 antibody (ab654), anti- β actin antibody (ab6276), anti- β 1 spectrin (ab2808), and anti-(α + β) spectrin (ab11182). Antibodies from Santa Cruz Biotechnology: anti-Ankyrin-1 (sc-12,733). Antibodies from Novus Biologicals: anti-protein 4.2 (NBP1-56,647). All other chemicals were highest purity grade available commercially.

### 2.2 Mice and Blood Samples from Mice

**Fig. 1:**
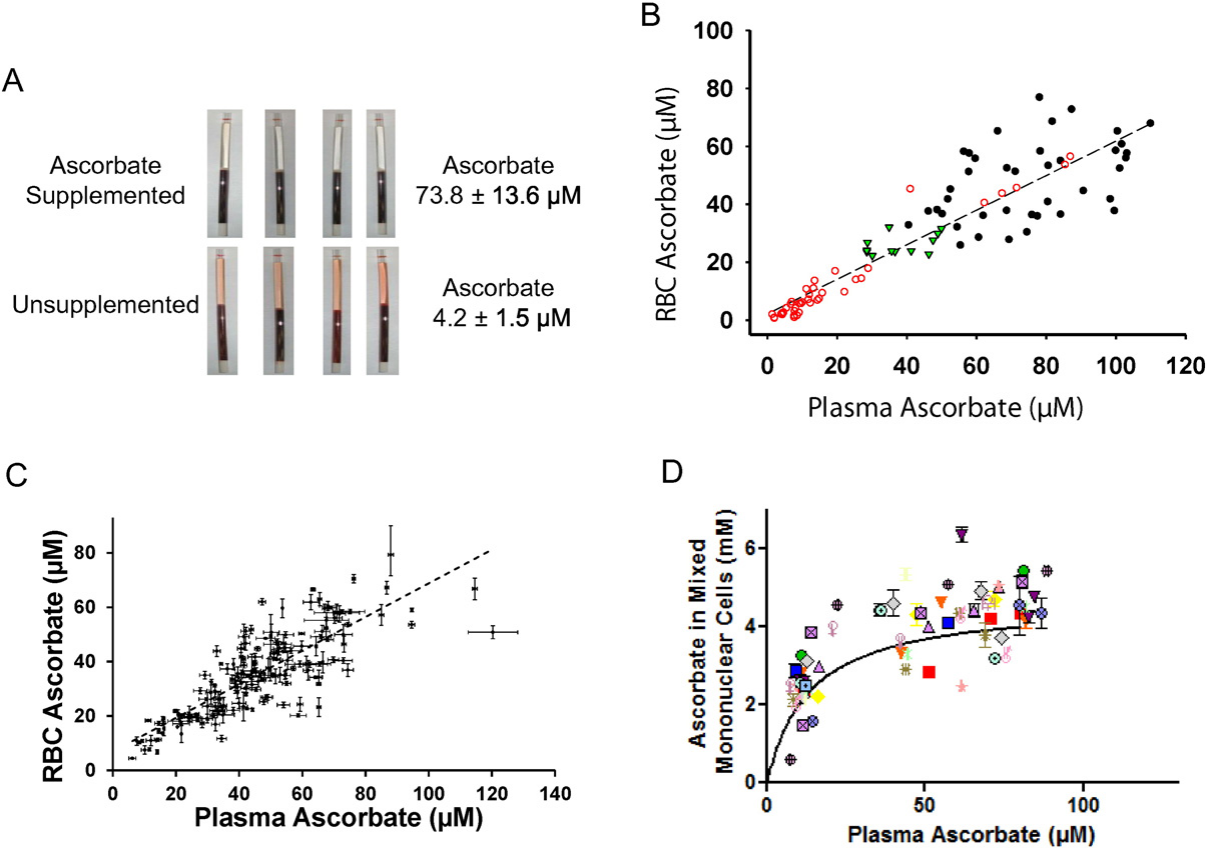
Hypothesis origin and vitamin C concentrations in RBCs and plasma. A. Hypothesis origins: findings from centrifugation of mouse whole blood. For ascorbate values, samples were obtained from 5 *Gulo*^*−*/*−*^ mice supplemented with ascorbate 1 g/L in drinking water for 12 weeks and 5 *Gulo*^*−*/*−*^ mice not supplemented with ascorbate for 12 weeks, plasma concentrations indicated in the panel. *Gulo*^*−*/*−*^ mice do not make ascorbate. When ascorbate was provided, mice received it via drinking water, which was changed daily. B. Mouse RBC ascorbate as a function of plasma ascorbate. 12 wildtype (WT) mice were unsupplemented for 14 weeks (); 10 *Gulo*^*−*/*−*^ mice were unsupplemented from 0 to 14 weeks (); and 5 *Gulo*^*−*/*−*^ mice were supplemented with 1 g/L ascorbate in drinking water from 0 to 17 weeks (●). Each symbol represents a separate blood sample. R = 0.91, p < 0.01. C. Human RBC ascorbate as a function of plasma ascorbate in 153 healthy blood donors, ascorbate measured by HPLC with coulometric electrochemical detection (Li et al., 2012). Each sample was measured in triplicate, samples without error bars indicate SD was less than symbol size. R = 0.82, p < 0.02. D. Human mononuclear cell ascorbate (all non-open circle symbols) as a function of plasma ascorbate, ascorbate measured by HPLC. In-patient healthy subjects (6 men and 13 women) who were at steady state for vitamin C doses of 30, 60, 100, 200, 400, 1000, and 2500 mg in two divided doses daily underwent apheresis with elutriation of cell-enriched product to obtain mixed mononuclear cells, as described (Levine et al., 1996, Levine et al., 2001). Each symbol represents a different subject. For each subject, samples were obtained at 1–5 different vitamin C doses at steady state for that dose (Levine et al., 1996, Levine et al., 2001).

Animal experiments were approved by the Animal Care and Use Committee NIDDK, NIH, and were conducted in accordance with NIH guidelines. Unless otherwise indicated, mice were 10–14 week old males with free access to food and water, and were maintained on regular chow diet (NIH-07) without detectable ascorbate (detection limit 10 nM). Mice were type C57BL/6 (wildtype, WT) (Charles River Laboratories, Wilmington, MA, USA); transgenic AZIP mice (original FVB/N A-ZIP/F-1 line) Moitra et al., 1998); and gulonolactone oxidase (*Gulo*^+/*−*^) mice (Mutant Mouse Regional Resource Center, University of California at Davis, USA), bred as described (Maeda et al., 2000). Plasma and RBCs were obtained from mouse whole blood as described (Li et al., 2012), with the modification that samples were centrifuged at 200 x g for 5 min due to hemolysis (see Fig. 1 and results). When ascorbate was provided, mice received it via drinking water, which was changed daily, or via gavage where indicated. See supplementary methods for additional details.

### 2.3 Human Subjects and Samples

Clinical research was approved by the Institutional Review Board, NIDDK/NIAMS, NIH, and conducted in accordance with NIH guidelines. Blood and cell samples from healthy subjects (NIH Protocols 04-DK-0021; 99-CC-0168; and 92-DK-0033) and diabetic subjects (NIH Protocol 04-DK-0021) were obtained and processed as described (Levine et al., 1996) (Li et al., 2012).

### 2.4 Erythrocyte Osmotic Fragility

RBC deformability is related to RBC osmotic fragility (Clark et al., 1983). RBC osmotic fragility based on resistance of RBCs to lysis as a function of decreasing NaCl concentration was performed as described (Parpart et al., 1947) with modifications (see supplementary methods).

### 2.5 Dehydroascorbic Acid and Ascorbate Transport

#### 2.5.1 Preparation of RBCs

RBCs were prepared as described (Li et al., 2012), with centrifugation modifications above.

#### 2.5.2 Transport of Dehydroascorbic Acid and Ascorbate into Mouse and Human RBCs

Human (50 μL) or mouse (30 μL) RBCs were added to PBS (450 μL for human RBCs, 270 μL for mouse RBCs) containing 5 mM glucose and freshly prepared ascorbate, [^14^C]ascorbate, dehydroascorbic acid, or [^14^C]dehydroascorbic acid. RBCs and supernatants were prepared and analyzed as described previously (Li et al., 2012).

### 2.6 Inhibition of 3-O-[^3^H]MG and [^14^C]DHA Uptake into Mouse and Human RBCs

#### 2.6.1 *Inhibition of 3-O-*[^*3*^*H*]*MG and* [^*14*^*C*]*DHA Uptake into Mouse and Human RBCs by Unlabeled* 3-O-*MG*

Human RBCs 2 mL prepared as above were incubated with 20 mM 3-O-MG in PBS (final volume 20 mL) for 20 min at 37 °C (See supplementary methods). After centrifugation at 500 x g for 5 min, the supernatant was removed and preloaded RBCs placed on ice until use. For competition between unlabeled and 3-O-[^3^H]MG, RBC 50 μL were added to 450 μL PBS on ice containing 1 μCi/mL 3-O-[^3^H]MG and one of the following concentrations of unlabeled 3-O-MG: 0.005, 1, 2.5, 5, 10, 20, 30 mM. For competition between unlabeled 3-O-MG, and [^14^C]DHA, RBC 50 μL were added to 450 μl PBS on ice containing freshly prepared 5 μM [^14^C]DHA and one of the following concentrations of unlabeled 3-O-MG: 0.005, 1, 2.5, 5, 10, 20, 30 mM. After incubation on ice for 1 min, 1 mL ice-cold stop buffer (10 μM cytochalasin B in PBS) was added, and the mixture centrifuged at 200 x g for 3 min at 4 °C. The supernatant was discarded, and RBCs were washed twice more at 4 °C. From the resulting RBC pellet, 40 μL were added to 360 μL ultrapurified water and the mixture repetitively pipetted for at least 10 s. The resulting lysate was transferred to a centrifugal filtration device (Amicon Ultra 0.5 ml 10 K Ultracell, Millipore) (Li et al., 2012), and centrifuged at 14,000 x g for 15 min at 4 °C. Filtrate 100 μL was added to 5 mL scintillation fluid followed by scintillation spectrometry analyses.

Mouse RBC experiments were conducted similarly, with the following modifications. For pre-loading RBCs with 3-O-MG, 0.4 mL washed RBCs were re-suspended in 3.6 mL PBS containing 20 mM 3-O-MG at 37 °C for 20 min, with removal of supernatant and placement on ice until use. For 3-O-MG competition experiments with 3-O-[^3^H]MG, 40 μL pre-loaded RBCs were added to 360 μL PBS at 37 °C containing 1 μCi/mL 3-O-[^3^H]MG with 5 μM or 30 mM unlabeled 3-O-MG. For 3-O-MG competition experiments with [^14^C] DHA, 40 μL pre-loaded RBCs were added to 360 μL PBS at 37 °C containing freshly prepared 5 μM [^14^C] DHA with 5 μM or 30 mM unlabeled 3-O-MG. After RBC addition, mixtures were incubated at 37 °C for 1 min, followed by addition of ice-cold stop buffer (PBS with 10 μM cytochalasin B), washing, lysis, filtration, and analyses by scintillation spectrometry.

#### 2.6.2 *Inhibition of 3-O-*[^*3*^*H*]*MG and* [^*14*^*C*]*DHA Uptake into Human and Mouse RBCs by Cytochalasin B*

After washed human RBCs 50 μL were re-suspended in 450 μL PBS with 20 mM 3-O-MG for 20 min at 37 °C as above, 0.5 μL of either dimethyl formamide alone or containing cytochalasin B was added. Final cytochalasin B concentration was 20 μM. After RBCs were incubated for an additional 15 min at 37 °C, they were pelleted by centrifugation at 50 x g for 5 min at 4 °C and 400 μL of supernatant was removed. The loose RBC pellet was re-suspended with 500 μL of pre-chilled PBS containing either 3-O-[^3^H]MG (5 μM, 1 μCi/mL) or [^14^C]dehydroascorbic acid (DHA)(freshly prepared, 5 μM, 0.027 μCi/mL), and incubated for 15, 30, 45 or 60 s on ice. At the end of the incubation time, 1 mL of prechilled PBS containing 20 mM 3-O-MG and 20 μM cytochalasin B was added, and RBCs were washed 3 times at 4 °C using this solution. As above, the final pellet was lysed in water, filtered using a centrifugal filtration device, and analyzed by scintillation spectrometry.

For mouse RBCs, experiments were conducted as for human RBCs with the following modifications: time point was one minute, temperature was 37 °C.

### 2.7 Xenopus Laevis Oocyte Transport Assay

cRNAs (complementary RNAs) of human *GLUT 1* or mouse *Glut 3* were prepared by *in vitro* transcription (mMessage mMachine, Ambion). *Xenopus laevis* oocytes were isolated and injected with cRNAs as described (Corpe et al., 2013), see supplementary methods for details.

### 2.8 Western Blot

For sample preparation, one volume of mouse or human intact red cells were washed three times with cold PBS before lysis using 4 volumes of modified RIPA buffer (50 mM Tris, pH 7.4, 150 mM NaCl, 0.1% SDS, 0.25% Na deoxycholate, 2% Triton-X100, 1 mM PMSF, 2uM leupeptin). Western blot was performed on RBC lysates according to manufacturer's instructions (Life Technologies, Carlsbad CA), see supplementary methods for details.

Anti-GLUTs (1–4) antibodies were all polyclonal antibodies. Based on supplier information, antibodies detected transporters from mice, rats, and humans. For GLUT Western blots of RBC lysates from human and mice: if no or weak bands were detected in either species, a positive control of the species (human/mouse cell lines or isolated mouse tissue) was added to determine whether the antibody recognized epitopes across species, see supplementary methods for details. GLUT 1 Western blots were developed using normal sensitivity substrate (Pierce ECL Western blot Substrate, Thermo), while other GLUT Western blots were developed using higher sensitivity substrate (SuperSignal West Pico Chemiluminescent Substrate, Thermo). RBC ghosts were prepared as described (Steck and Kant, 1974). For mouse RBCs, anti- β 1 spectrin antibody was used to detect β-spectrin, and anti-(α + β) spectrin antibody was used to detect α-spectrin. For human RBCs, anti-(α + β) spectrin antibody was used to detect β-spectrin and α-spectrin.

### 2.9 Confocal Microscopy

RBCs were fixed using 0.1% glutaraldehyde, stained with Alexa Fluor 488 phalloidin (5 units/mL), and analyzed by confocal microscopy (Yau et al., 2012). Confocal laser scanning microscopy analysis was performed with a Duo-Scan system (LSM 5 LIVE; Carl Zeiss Inc., Thornwood, NY) using a 63 × 1.4 NA oil immersion objective. Laser 489 nm line was applied for excitation and emission was collected with 518 nm filter. Images were acquired using ZEN 2007 software (Carl Zeiss, Inc.). Whole cell and biconcave area diameters were determined by drawing a horizontal line through the center of each target RBC and calculating the distances between two points where fluorescent intensities were most different.

### 2.10 Ascorbic Acid, Dehydroascorbic Acid, and Glucose Analyses

Whole blood glucose was measured by established methods, see supplementary methods for details. Briefly, mouse whole blood glucose measurements were based on conversion of glucose to gluconolactone (Accu Chek, Roche Diagnostics), and humans samples were measured by the hexokinase method (Sacks, 2008). Values between methods used for mouse and human samples are within 10% (Freckmann et al., 2012). Ascorbic acid and dehydroascorbic acid were measured by HPLC (high performance liquid chromatography) as described (Li et al., 2012). RBC intracellular water space was 70% of packed RBC volume (Kageyama et al., 1989, Mendiratta et al., 1998). Although RBC volume differs from mice to humans, mice have similar intracellular water space (Tanabe et al., 1986). White blood cell volumes, determined as previously described, are 0.25 μL/10^6^ mixed mononuclear cells (Bergsten et al., 1990).

### 2.11 Calculations

Data displayed are mean ± S.D. unless otherwise indicated. Numbers of patients or mice are indicated in legends. Otherwise, each data point represents N of at least 3 replicates unless noted differently in figure legends. Points lacking error bars indicate S.D. was less than symbol size. Comparisons between 3 or more groups utilized 1-way ANOVA followed by Tukey's multiple comparison test (Graphpad Prism version 5.01; SigmaPlot version 13). Two groups were compared using, 2-tailed Student's *t* test (Excel 2010). p ≤ 0.05 or less was considered significant.

Significance versus respective control was indicated as follows: *: p ≤ 0.05; **: p ≤ 0.01; ***: p ≤ 0.005; ****: p < 0.001.

## 3 Results

### 3.1 Hypothesis Origin and Relevant Vitamin C Concentrations in Mice and Humans

The original clue linking ascorbate, diabetes and RBCs came from unexpected observations of whole blood obtained from mice (*Gulo*^*−*/*−*^) unable to synthesize vitamin C. When whole blood was centrifuged, samples from unsupplemented mice had visible hemolysis (Fig. 1A). With slower centrifugation for longer times to prevent hemolysis, plasma ascorbate concentrations could be determined. Ascorbate values for supplemented *Gulo*^*−*/*−*^ mice were ~ 74 μM, and for unsupplemented *Gulo*^*−*/*−*^ mice were ~ 4 μM. Hemolysis only in samples with low plasma ascorbate concentrations implicated low vitamin C within RBCs as a cause. To explore this possibility, we directly measured mouse RBC vitamin C concentrations, and when compared to plasma concentrations from the same whole blood samples, a linear relationship was found (R = 0.91, p < 0.01) (Fig. 1B).

To learn whether mouse vitamin C data were relevant to humans, we measured human plasma and RBC ascorbate concentrations (Fig. 1C). In whole blood from > 150 random healthy humans, RBC ascorbate concentration was at or below the associated plasma concentration, with a linear relationship between both compartments (R = 0.82, p < 0.02). In > 10% of subjects, RBC ascorbate values were < 20 μM, in the early vitamin C deficiency range (Levine et al., 1996, Levine et al., 2001). The concentrations in RBCs were then compared to another human circulating cell type, mononuclear cells (Fig. 1D). In contrast to RBCs (Fig. 1C), mononuclear cells had ascorbate concentrations that were > 30-fold higher than concurrent plasma concentrations (Fig. 1D). Mononuclear cell data are consistent with other cell-types (Levine et al., 1996, Levine et al., 2001).

### 3.2 Osmotic Fragility in Mouse RBCs in Relation to Ascorbate Concentrations and Underlying Mechanism

**Fig. 2:**
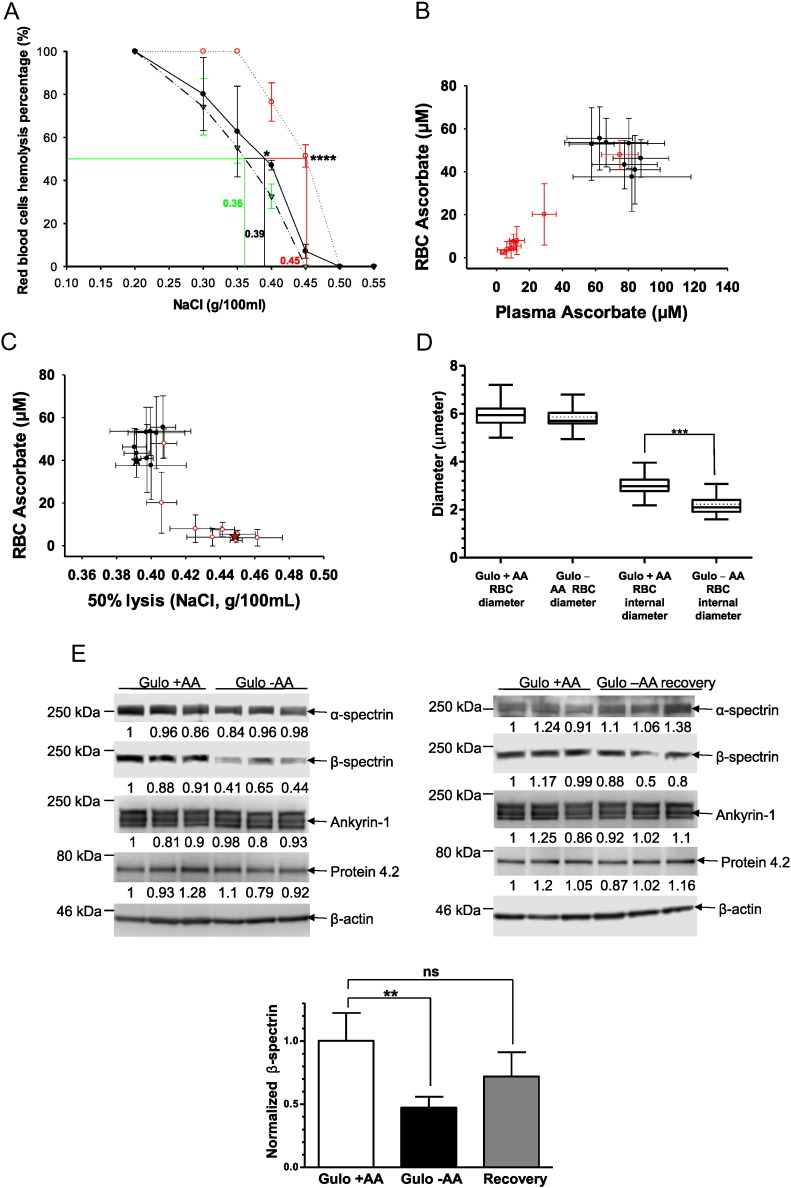

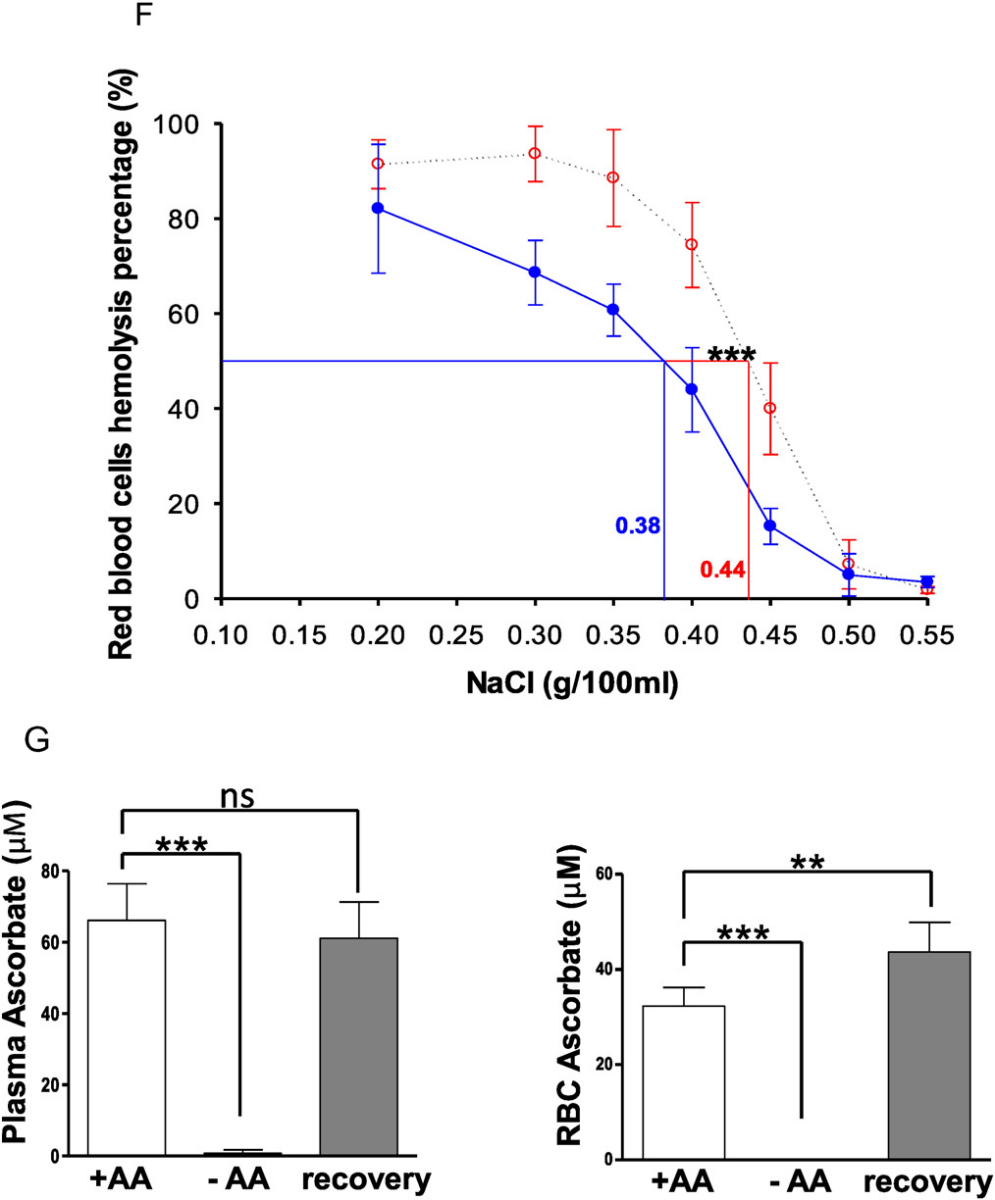
Osmotic fragility in mouse RBCs as a function of their ascorbate concentrations and underlying mechanism. A. Osmotic RBC fragility in mouse RBCs as a function of NaCl concentration. RBC samples were obtained from 5 WT unsupplmented mice (); 5 *Gulo*^*−*/*−*^ mice without ascorbate for 12 weeks (); and 5 *Gulo*^*−*/*−*^ mice supplemented with 1 g/L ascorbate for 12 weeks (●); N = 5 mice per point. The 50% lysis point for each condition (horizontal line) is determined by the vertical line of the same color. Plasma ascorbate concentrations: WT mice, 77.6 μM; *Gulo*^*−*/*−*^ mice, 4.0 μM; *Gulo*^*−*/*−*^ mice supplemented with 1 g/L ascorbate for 12 weeks 64.8 μM. Statistics (two-tailed T-test) for 50% lysis: WT vs. unsupplemented *Gulo*^*−*/*−*^ mice, p < 0.001; unsupplemented vs supplemented *Gulo*^*−*/*−*^ mice, p < 0.05. Wildtype and supplemented *Gulo*^*−*/*−*^ mice were not statistically different. B. RBC ascorbate as a function of plasma ascorbate in *Gulo*^*−*/*−*^ mice also tested for RBC osmotic fragility (see Fig. 2C). Five *Gulo*^*−*/*−*^ mice were supplemented with ascorbate 1 g/L for 17 weeks. Ten *Gulo*^*−*/*−*^ mice were deprived of ascorbate for 0–14 weeks, followed by gavage supplementation with 0.2 mg of ascorbate in 100 μL water once at 14 weeks and once at 17 weeks. Gavage was performed to maintain mice at low ascorbate values while preventing demise from scurvy (see supplementary methods). N = 5 mice per point. Samples from ascorbate unsupplemented mice indicated by (); samples from ascorbate supplemented mice indicated by (●). Individual data points are also included in Fig. 1B. C. RBC osmotic fragility in *Gulo*^*−*/*−*^ mice as a function of RBC ascorbate. 50% RBC lysis (see Fig. 2A) was tested in relation to RBC ascorbate concentration from 5 *Gulo*^*−*/*−*^ mice supplemented with ascorbate 1 g/L for 17 weeks or 10 *Gulo*^*−*/*−*^ mice deprived of ascorbate for 0–14 weeks, and then supplemented by gavage with 0.2 mg of ascorbate in 100 μL water once at 14 weeks and once at 17 weeks (see Fig. 2B and supplementary methods). N = 5 mice per point. Star symbols represent 50% lysis of RBCs from *Gulo*^*−*/*−*^ mice unsupplemented (red) or supplemented (black) with ascorbate from Fig. 2A. Samples from ascorbate unsupplemented mice indicated by (); samples from ascorbate supplemented mice indicated by (●). D. Biconcave and total diameters of RBCs from ascorbate supplemented and unsupplemented *Gulo*^*−*/*−*^ mice. Ascorbate supplemented *Gulo*^*−*/*−*^ mice (N = 6) received ascorbate 1 g/L for 12 weeks, and unsupplemented mice (N = 6) had no ascorbate for 12 weeks (plasma ascorbate concentrations were 66 ± 10 and 1 ± 1 μM respectively, see Fig. 2G). Forty to fifty RBCs in full view orientation using confocal microscopy were randomly selected from each group for measurement of diameters. ***: p < 0.0001 (t-test). See supplementary Fig. 1B for images. E. β-Spectrin in lysates of mouse RBCs. RBC lysates from ascorbate supplemented, unsupplemented, and unsupplemented and then resupplemented *Gulo*^*−*/*−*^ male mice were prepared as described in methods. Lysates were analyzed by Western blots using antibodies to α-spectrin, β-spectrin, ankyrin-1, protein 4.2 and β-actin. β-actin was used as loading control, and triplicates are displayed. Densitometry analyses are below each band. Left panel: *Gulo*^*−*/*−*^ supplemented vs. unsupplemented mice. Right panel: *Gulo*^*−*/*−*^ supplemented mice vs. gulo^−/−^ mice that were unsupplemented and then resupplemented for 10 days (labeled Gulo ascorbate recovery). In lower panel, β-spectrin in mouse RBCs was normalized to β-actin for protein band signal intensities, using data from control, depleted and repleted *Gulo*^*−*/*−*^ mice in two upper panels. Plasma ascorbate concentrations were: 66 ± 10 (supplemented mice, N = 6); 1 ± 1 μM (unsupplemented mice for 12 weeks, N = 6); and 61 ± 9 μM (unsupplemented mice for 12 weeks then resupplemented for 10 days, N = 6). Mice were resupplemented with ascorbate 2 g/L in drinking water to ensure rapid supplementation. F. Osmotic RBC fragility as a function of NaCl concentration. *Gulo*^*−*/*−*^ mice were deprived of ascorbate for 12 weeks (), and the same mice were supplemented with ascorbate 2 g/L in drinking water for the following 10 days (). N = 5 mice per point. The 50% lysis point for each condition (horizontal line) is determined by the vertical line of the same color. Plasma ascorbate concentrations (see supplementary Fig. 1C): *Gulo*^*−*/*−*^ mice without ascorbate, 1.0 ± 1 μM; *Gulo*^*−*/*−*^ mice supplemented with 2 g/L ascorbate for 10 days, 61 ± 9 μM. For unsupplemented vs. resupplemented RBC 50% lysis: p < 0.005 (two tailed t-test). G. Ascorbate concentrations in plasma and RBCs from *Gulo*^*−*/*−*^ male mice. Mice were continuously supplemented (N = 5); unsupplemented (N = 8); and unsupplemented then resupplemented (N = 6). Unsupplemented *Gulo*^*−*/*−*^ mice did not receive ascorbate for 12 weeks. Resupplemented mice received ascorbate in drinking water 2 g/L for 10 days. Resupplemented mice had previously not had ascorbate for 12 weeks.

Based on Fig. 1, low RBC vitamin C concentrations were clinically plausible. Next steps were to investigate RBC fragility in relation to plasma and RBC vitamin C concentrations. Because low ascorbate levels are not easily obtainable in humans (Levine et al., 1996, Levine et al., 2001), we studied *Gulo*^*−*/*−*^ mice (Maeda et al., 2000). We predicted that plasma and RBC ascorbate concentrations in *Gulo*^*−*/*−*^ mice would be a function of ascorbate depletion or supplementation, and it would be possible to have animals with low or high values. Wildtype (WT) mice, which synthesize ascorbate, were used as controls. RBC osmotic fragility was a surrogate for RBC deformability (Clark et al., 1983). RBCs were obtained from ascorbate unsupplemented *Gulo*^*−*/*−*^ mice with low plasma ascorbate concentrations (4 μM). These RBCs were more sensitive to osmotic lysis compared to supplemented *Gulo*^*−*/*−*^ mice with normal plasma ascorbate concentrations (64 μM) (p < 0.05 for osmotic lysis) and compared to WT mice, with plasma ascorbate concentrations of 77 μM (p < 0.001 for osmotic lysis) (Fig. 2A). To further characterize osmotic fragility, it was necessary to use RBCs whose ascorbate concentrations varied over a wide range. This was achieved by using *Gulo*^*−*/*−*^ mice and withholding ascorbate for as long as 14 weeks (Fig. 2B, see Fig. 2 legend for details). The longer *Gulo*^*−*/*−*^ mice remained unsupplemented, the more plasma and RBC ascorbate values declined (supplementary Fig. 1A). Osmotic fragility was then tested in relation to these declining RBC ascorbate values (Fig. 2C). Once RBC values were < 10 μM, increased osmotic fragility occurred. These data, as in Fig. 2A, indicate that RBC fragility is inversely related to RBC ascorbate concentration.

To get clues about osmotic fragility induced by ascorbate deficiency, we examined RBCs from ascorbate repleted and depleted *Gulo*^*−*/*−*^ mice by confocal microscopy. A decreased surface-to-volume ratio indicates swelling, and internal diameters of RBCs are an inverse function of swelling because swelling produces a decreased surface-to- volume ratio (Diez-Silva et al., 2010). A decreased surface to volume ratio would be expected to decrease deformability, which was observed (Fig. 2D). Internal diameters (biconcave diameters) of RBCs were measured using confocal microscopy, and compared to controls, ascorbate-deficient RBCs had significantly lower internal diameters and a spherocyte-like appearance (Fig. 2D, supplementary Fig. 1B). The spherocyte appearance in these mouse RBCs was similar to RBCs from humans with mild hereditary spherocytosis, one of the few clinical disorders in which RBCs have increased osmotic sensitivity. Given the similarities in RBC appearance, and because human hereditary spherocytosis may be due to defects in spectrins or ankyrin or both, we used RBCs from *Gulo*^*−*/*−*^ mice to quantitate these proteins by Western blot. *Gulo*^*−*/*−*^ mice were ascorbate supplemented, ascorbate depleted, or ascorbate depleted followed by re-supplementation. Ankyrin-1 and α-spectrin were unchanged, but β-spectrin was significantly less in RBCs from depleted *Gulo*^*−*/*−*^ male mice compared to controls (Fig. 2E left side). Depleted *Gulo*^*−*/*−*^ male mice were repleted with ascorbate for 10 days, and RBCs were re-studied by Western blot (Fig. 2E right side); osmotic sensitivity (Fig. 2F); and confocal microscopy (supplementary Fig. 1C). β-Spectrin in RBCs from depleted then re-supplemented *Gulo*^*−*/*−*^ mice was similar to β-spectrin in RBCs from continuously supplemented *Gulo*^*−*/*−*^ mice (Fig. 2E right side). Western blot findings for β-spectrin are summarized in Fig. 2E (lower panel). Osmotic fragility measurements indicated that compared to controls, ascorbate depleted mice had increased fragility, which was reversed with ascorbate supplementation (Fig. 2F). RBC appearance in depleted then re-supplemented *Gulo*^*−*/*−*^ mice was indistinguishable from controls (supplementary Fig. 1C). Ascorbate values in plasma and RBCs confirm supplementation, depletion, and repletion (Fig. 2G). Other than described above, RBCs from ascorbate supplemented and deficient male mice had indistinguishable hematologic parameters (supplementary Fig. 1D). Using female *Gulo*^*−*/*−*^ mice, we evaluated ankyrin-1 and β-spectrin in ascorbate supplemented and unsupplemented mice; and in ascorbate depleted mice that were then re-supplemented for 3 days (supplementary Fig. 1E-G). Consistent with the findings using RBCs from male *Gulo*^*−*/*−*^ mice, the data showed that only β-spectrin declined, that the decline reversed within as short a time as 3 days, and that ascorbate depletion and repletion values in plasma and RBCs were as predicted. Additionally, acute *in vitro* supplementation of RBCs from ascorbate deficient *Gulo*^*−*/*−*^ mice did not reverse the decline in β-spectrin that was a consequence of low RBC ascorbate (supplementary Fig. 1H).

### 3.3 Potential Relationship to Diabetes: Dehydroascorbic Acid and Ascorbic Acid Transport into Human and Mouse RBCs

**Fig. 3:**
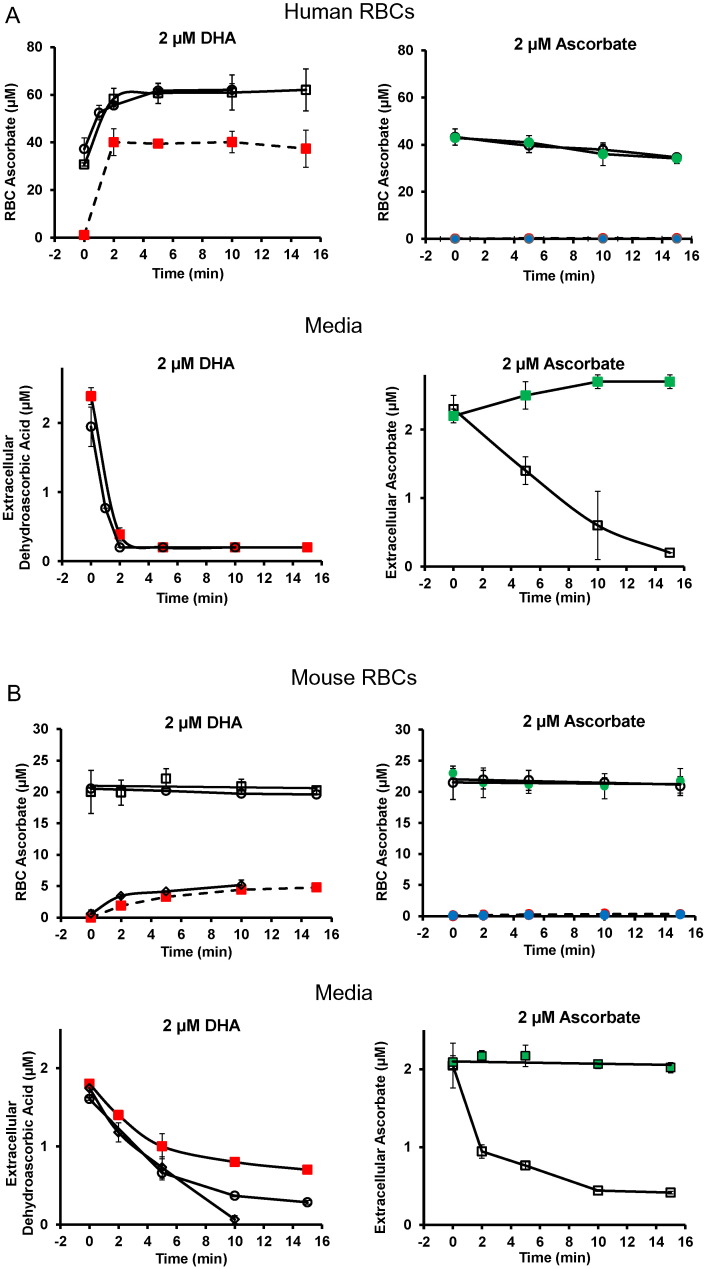
Dehydroascorbic acid and ascorbic acid transport into human and mouse RBCs as a function of time. Humans RBCs are in grouped Figure A, mouse RBCs are in Figure B. Each grouped figure contains four panels. For each grouped figure: in the left upper panel, RBC ascorbate concentration is shown after incubation with one concentration of dehydroascorbic acid; and in the right upper panel, RBC ascorbate concentration is shown after incubation with the same concentration of ascorbic acid. The lower panels are the corresponding extracellular concentrations: dehydroascorbic acid added on the left lower side, ascorbic acid added on the right lower side. Within each panel, a different symbol represents blood from a different human or mouse, and from each human or mouse three or more independent samples were obtained and analyzed at every time point. All open black and white symbols on both sides indicate sample analyses by HPLC, with no reducing agent added. For color symbols: green symbols (, ) indicate analyses by HPLC, with reducing agent (500 μM TCEP) present during incubation; red symbols (, ) indicate analyses by scintillation spectrometry, without reducing agent added; blue symbols (, ) indicate analyses by scintillation spectrometry, with reducing agent (500 μM TCEP) present during incubation. The concentration of dehydroascorbic acid/ascorbic acid was 2 μM. For clarity, concentrations and species are shown in each panel. Additional experiments with concentration of 5 μM is shown in supplementary figures. For human RBC experiments, 50 μL RBCs were incubated in 0.5 mL total volume using PBS with 5 mM glucose. For mouse RBC experiments, 30 μL RBCs were incubated in 0.3 mL total volume using PBS with 5 mM glucose. Note that initial concentrations are different in the same figures because animals/human often do not have the same initial values, and some data represent scintillation spectrometry results, where initial values should be close to or at zero. See methods and supplementary Fig. 3 legend for other details.

The potential connection between ascorbate, RBCs, and diabetes is that the probable species transported into RBCs is oxidized ascorbate, or dehydroascorbic acid (DHA), which enters via glucose transporters and then is immediately reduced intracellularly (Hughes and Maton, 1968, Mann and Newton, 1975, Bianchi and Rose, 1986, Mendiratta et al., 1998). To delineate the transported species in human and mouse RBCs, we used estimated physiologic plasma concentrations of DHA, which are 1–5% of ascorbate (Dhariwal et al., 1991, Lykkesfeldt, 2000); physiologic plasma ascorbate concentrations (Levine et al., 1996, Levine et al., 2001); freshly synthesized DHA (Rumsey et al., 1997, Corpe et al., 2013); and a recently developed assay for RBC ascorbate (Li et al., 2012). Extracellular DHA as substrate was measured to verify its presence (see Fig. 3 and supplementary Fig. 2). In human RBCs, DHA at 2 μM was avidly transported, such that within 2 min nearly all DHA moles in media were translocated into RBCs and reduced to ascorbate (Fig. 3A, supplementary Fig. 3 legend). No ascorbate was transported under the same conditions (Fig. 3A). Using DHA and ascorbate concentrations of 5 μM (supplementary Fig. 3A,) and human RBCs, similar findings were obtained: only DHA was transported. Mouse RBCs also transported only DHA and not ascorbate, but transport rates were 10–20 fold lower compared to human RBCs (Fig. 3B; supplementary Fig. 3B). Only ascorbate was found in RBCs (Li et al., 2012) (supplementary Fig. 4A,B).

### 3.4 Glucose Inhibition of DHA Uptake into RBCs *in vitro*, *GLUT* Transporters Expressed, and Glucose Effects on RBC Ascorbate Concentrations and Osmotic Fragility *in vivo*

**Fig. 4:**
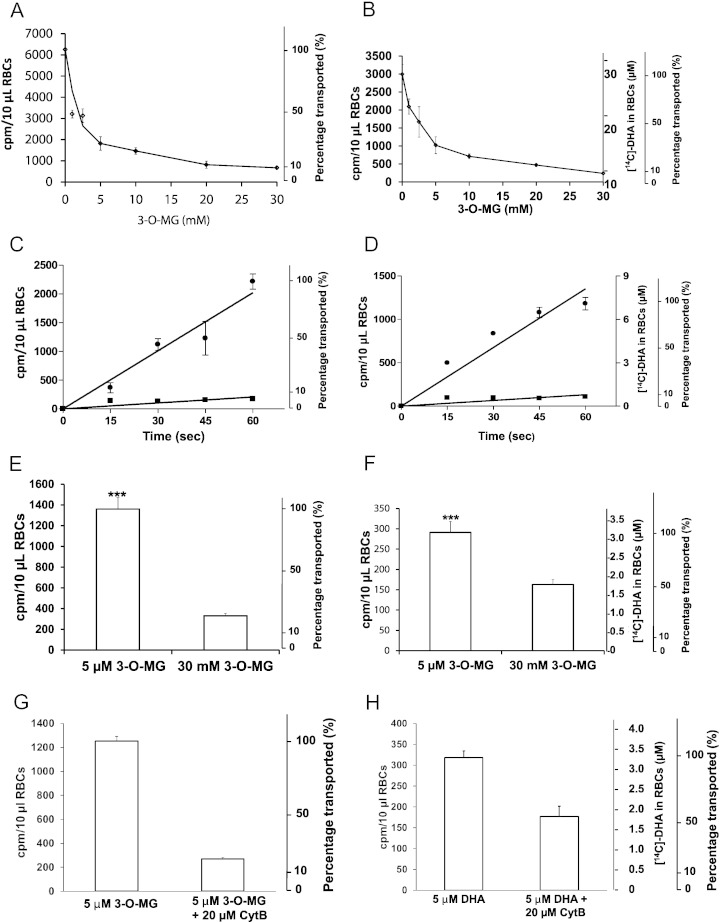
Glucose Inhibition of DHA uptake into RBCs *in vitro*; glucose effects on ascorbate concentrations in RBCs in vivo*.* A, B. Inhibition of 3-O-[^3^H]MG and [^14^C]DHA uptake into human RBCs by unlabeled 3-O-MG. After loading with 20 mM 3-O-MG at 37 °C, human RBCs 10% were washed and incubated on ice for 1 min with 0–30 mM unlabeled 3-O-MG as indicated and with either 5 μM 3-O-[^3^H]MG as control (A) or 5 μM [^14^C]DHA (B). Uptake of 3-O-MG and DHA were determined by scintillation spectrometry. C,D. Inhibition of 3-O-[^3^H]MG and [^14^C]DHA uptake into human RBCs by cytochalasin B (CytB). After loading with 20 mM 3-O-MG at 37 °C and without washing, human RBCs 10% were incubated on ice for 1 min with (■) or without (●) 20 μM cytochalasin B and with 5 μM 3-O-[^3^H]MG (C) or with 5 μM [^14^C]DHA (D). E,F. Inhibition of DHA uptake into WT mouse RBCs by 3-O-MG. After loading with 20 mM 3-O-MG, mouse RBCs 10% were washed and incubated at 37 °C for 1 min. For control experiments (E), 5 μM 3-O-[^3^H]MG was added alone or with 30 mM 3-O-MG. For DHA uptake experiments (F), 5 μM [^14^C]DHA was added alone or with 30 mM 3-O-MG. G,H.  Inhibition of DHA uptake into WT mouse RBCs by cytochalasin B. After loading with 20 mM 3-O-MG, mouse RBCs 10% were washed and incubated at 37 °C for 1 min. For control experiments (G), 5 μM 3-O-[^3^H]MG was added alone or with 20 μM cytochalasin B. For DHA uptake experiments (H), 5 μM [^14^C]DHA was added alone or with 20 μM cytochalasin B.

Since DHA is exclusively transported, and the likely transporter is a facilitated glucose transporter (*GLUT 1*, *Glut 3*) (Vera et al., 1993, Rumsey et al., 1997, Corpe et al., 2013), we investigated DHA transport inhibition by the non-metabolized analog 3-O-methylglucose, using human RBCs. As essential control, [^3^H]3-O-methylglucose 5 μM uptake was inhibited by increasing concentrations of unlabeled 3-O-methylglucose 1–30 mM (Fig. 4A; supplementary Fig. 5A-E). Under the same conditions, 5 μM [^14^C]DHA uptake was inhibited by 1–30 mM 3-O-methylglucose in a matching pattern (Fig. 4B). DHA was fully reduced to ascorbate under these experimental conditions (supplementary Fig. 6). To account for transactivation and to maximize transport, RBCs were pre-loaded with 3-O-methylglucose (Stein, 1986, Carruthers and Naftalin, 2009, Carruthers et al., 2009, Sage and Carruthers, 2014). The glucose transporter inhibitor, cytochalasin B, inhibited uptake of 3-O-methylglucose and DHA nearly identically in human RBCs (Fig. 4C,D). With mouse RBCs, 30 mM unlabeled 3-O-methylglucose inhibited [^3^H]3-O-methylglucose and DHA uptake, although when compared to human RBCs, inhibition of DHA uptake by 3-O-MG was less robust (Fig. 4E,F). Similar findings in mouse RBCs were obtained with cytochalasin B as an inhibitor of [^3^H]3-O-methylglucose and DHA uptake: when compared to human RBCS, inhibition of DHA uptake by cytochalasin B was less robust (Fig. 4G,H). Based on Fig. 4, human RBCs transported DHA and 3-O-MG similarly, while mouse RBCs had a higher rate of 3-O-MG transport than DHA. Slower transport of DHA in mouse RBCs compared to human RBCs is consistent with the data in Fig. 3.

**Fig. 5:**
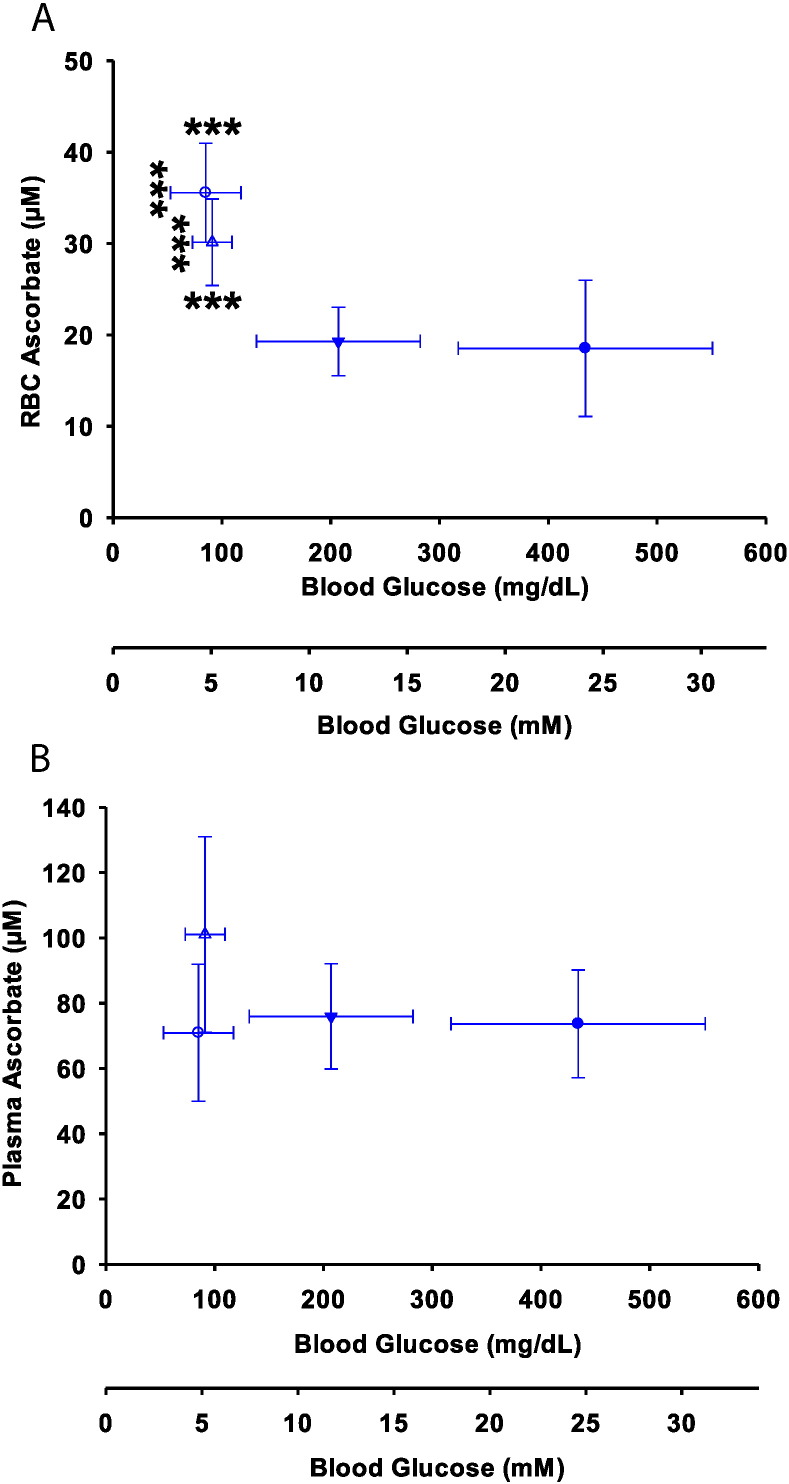
RBC and plasma ascorbate in mice in relation to glycemia. A. RBC ascorbate as a function of blood glucose in fed and fasted control (, ) and fed and fasted diabetic AZIP mice (, ). AZIP mice and control littermates were fed chow ad libitum until 16 h before sacrifice. Blood was withdrawn from mice that were fed or fasted 16 h and RBC ascorbate measured by HPLC. AZIP mice were tested together with littermate controls. B. Ascorbate plasma concentrations as a function of blood glucose in fed () or fasted AZIP () mice and fed () or fasted () WT controls. Control and AZIP mice synthesize ascorbate and do not have an intake requirement for it. There was no significant difference between plasma ascorbate values in fasted AZIP mice compared to fasted WT control mice.

Because transport of DHA and its inhibition were less robust in mouse RBCs *in vitro* compared to human RBCs *in vitro*, it was uncertain whether glucose would affect RBC ascorbate concentrations in mouse RBCs *in vivo*. To investigate, we used the AZIP lipoatrophic diabetes mouse model (Moitra et al., 1998). These mice have stable hyperglycemia that reverses overnight with fasting. RBC ascorbate and blood glucose, measured in non-fasted and fasted AZIP and WT littermate-control mice, displayed an inverse relationship (Fig. 5A). The decrease in RBC ascorbate induced by hyperglycemia occurred between 100 and 200 mg/dl glucose (5.5–11.1 mM), a key range in diabetes (American Diabetes Association: Position Statement, 2014, American Diabetes Association: Position Statement, 2015). To learn whether changes in ascorbate plasma values could explain the inverse relationship between plasma glucose and RBC ascorbate, plasma ascorbate values were measured as controls in all groups (Fig. 5B). The findings showed that there were no statistically significant changes in plasma ascorbate concentrations in normal and AZIP mice as a function of plasma glucose concentrations. Therefore, plasma ascorbate concentrations did not account for the observed relationship between glucose and RBC ascorbate. As a corollary, we investigated whether ascorbate affected glucose values in ascorbate supplemented and depleted *Gulo*^*−*/*−*^ mice. The values were not statistically different: 162 ± 14 mg/dl (9 ± 0.7 mM) vs 168 ± 21 mg/dl (9.3 ± 1.2 mM), respectively, N = 6 per group.

**Fig. 6:**
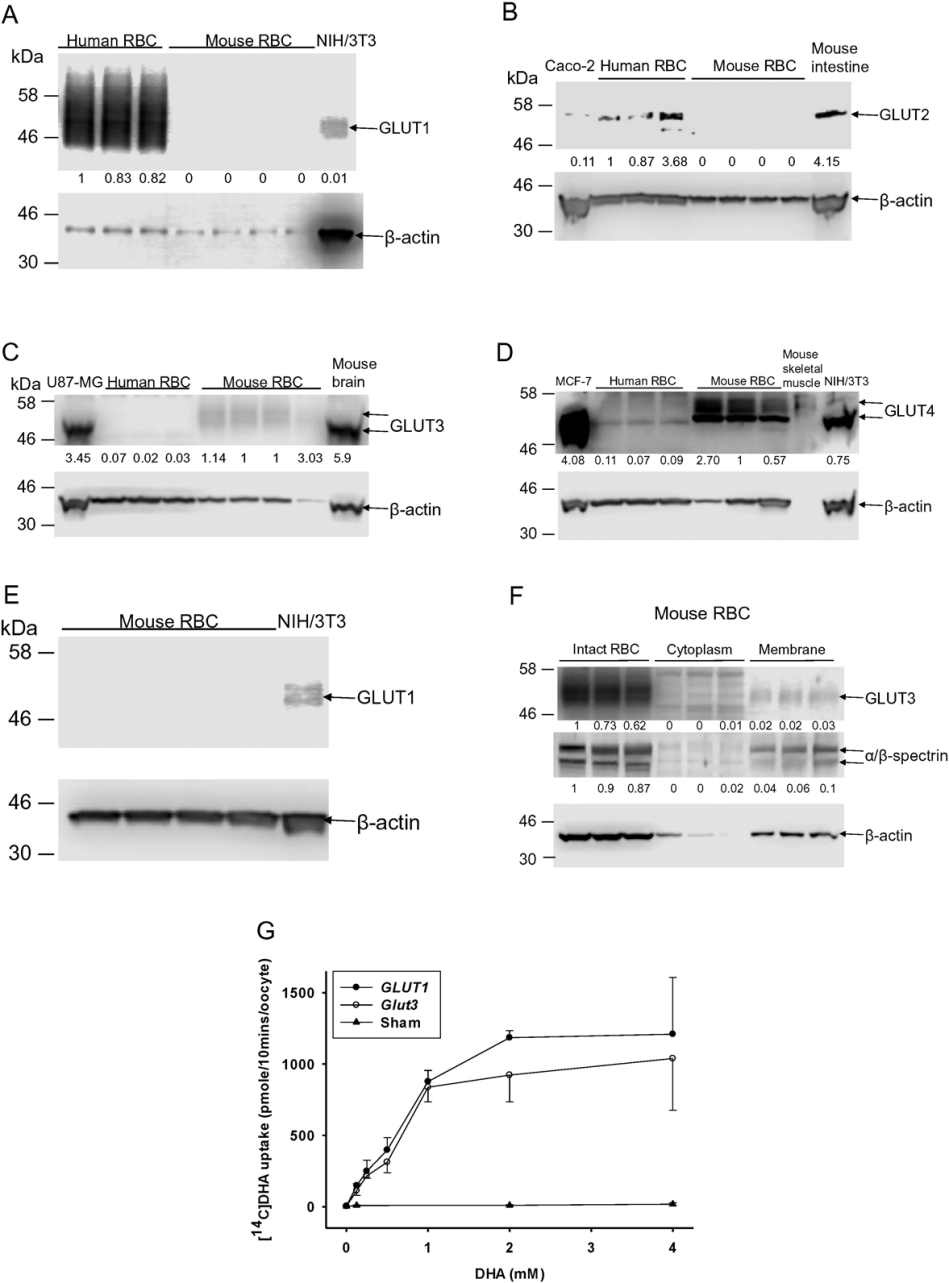
*GLUTs* in mouse and human RBCs and relevant transport activities. A-F. *GLUTs* in mouse and human RBCs as determined by Western blot. For *GLUT 1* analyses, 2.5 μL of human or mouse RBC lysate (RBC 5% *v*/*v*) and 20 μg of NIH/3 T3 cell lysate were analyzed. For mouse *Glut 1* and *Glut 2*, *3* and *4* analyses: 10 μL of lysate were analyzed from RBCs (RBC 20% *v*/*v*), cells (NIH/3 T3, Caco-2, U87-MG or MCF-7) or tissues (mouse intestine, brain or skeletal muscle). β-actin was used as loading control in each blot, and α/β spectrin were used to indicate RBC membrane separation. RBC ghosts were prepared as described (Steck and Kant, 1974). G. DHA uptake in injected *Xenopus laevis* oocytes. Mouse *Glut 3* and human *GLUT 1* cRNAs were injected and expressed in *Xenopus laevis* oocytes, as described (Rumsey et al., 1997). cRNA concentrations were 1 ng/nL. Sham-injected oocytes were injected with 36.8 nL of water. Each point is mean value of 10 oocytes ± S.D.

Because DHA transport rates are 10–20 fold different in mouse and human RBCs, and given conflicting data for transporter activities (Montel-Hagen et al., 2008b, Sage and Carruthers, 2014), we characterized *GLUTs* in these cells (Fig. 6A-G). Human RBCs expressed *GLUT 1* dominantly, with trace expression of *GLUTs 2–4*, generally consistent with prior findings (Concha et al., 1997). Mouse RBCs expressed *Gluts* 3 and 4 but not *Glut 1*, even when a high sensitivity method was used (Fig. 6E). Transporter activity data indicate that *GLUTs* 1 and 3 are by far the most robust DHA transporters (Rumsey et al., 1997, Rumsey et al., 2000, Corpe et al., 2013). Using *Xenopus* oocytes, we show here that human *GLUT 1* and mouse *Glut 3* transported DHA similarly (Fig. 6G). Considered together, the data suggest that *GLUT 1* is the dominant DHA transporter in human RBCs, while mouse RBCs utilize *Glut 3*, and perhaps *Glut 4*. Although mouse *Glut 3* and human *GLUT 1* had similar transport characteristics, there was substantially less *Glut 3* in mouse RBCs compared to *GLUT 1* in human RBCs (Fig. 6A, C). These findings are consistent with slower DHA transport in mouse RBCs compared to human RBCs (see Fig. 3, Fig. 4).

**Fig. 7:**
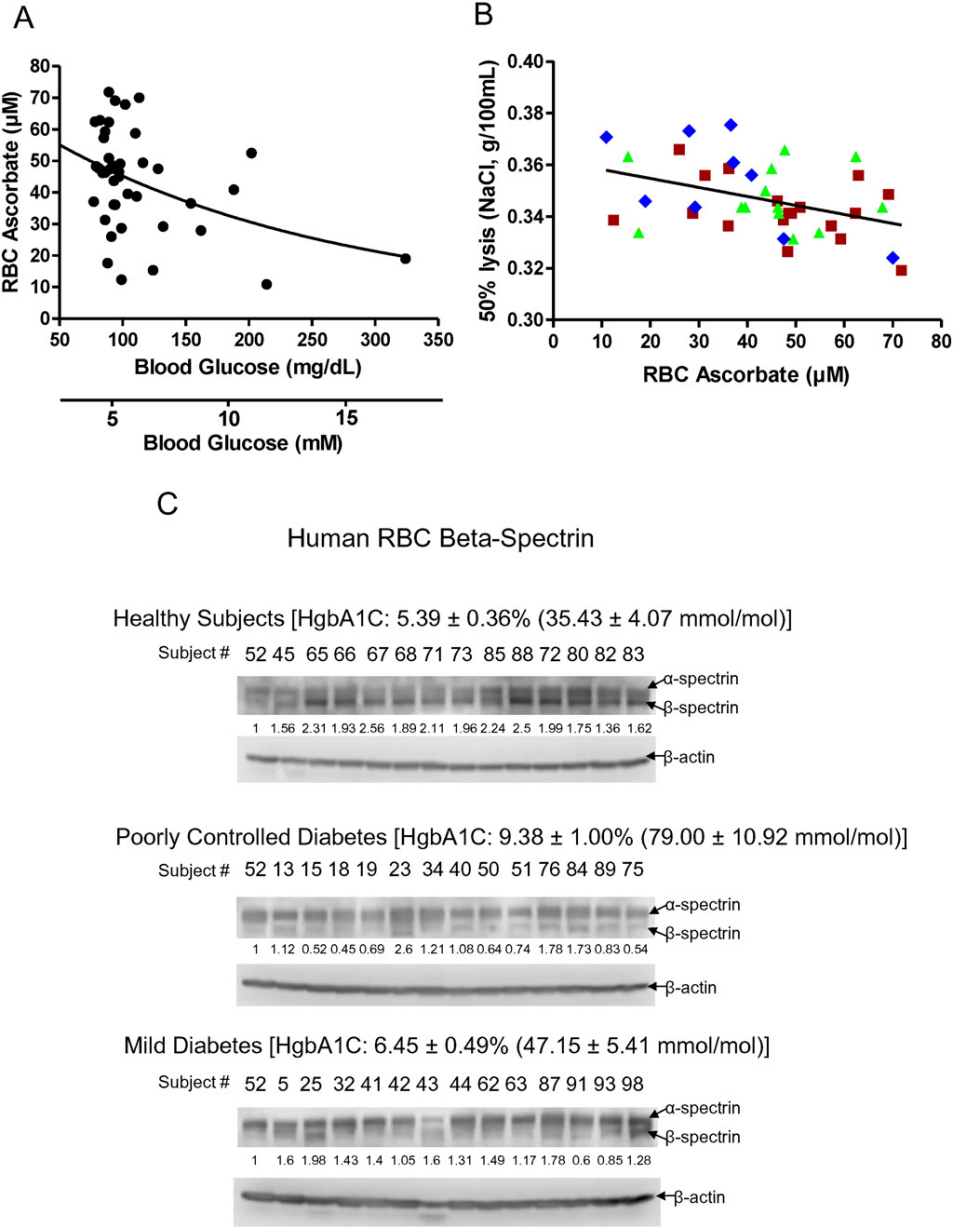

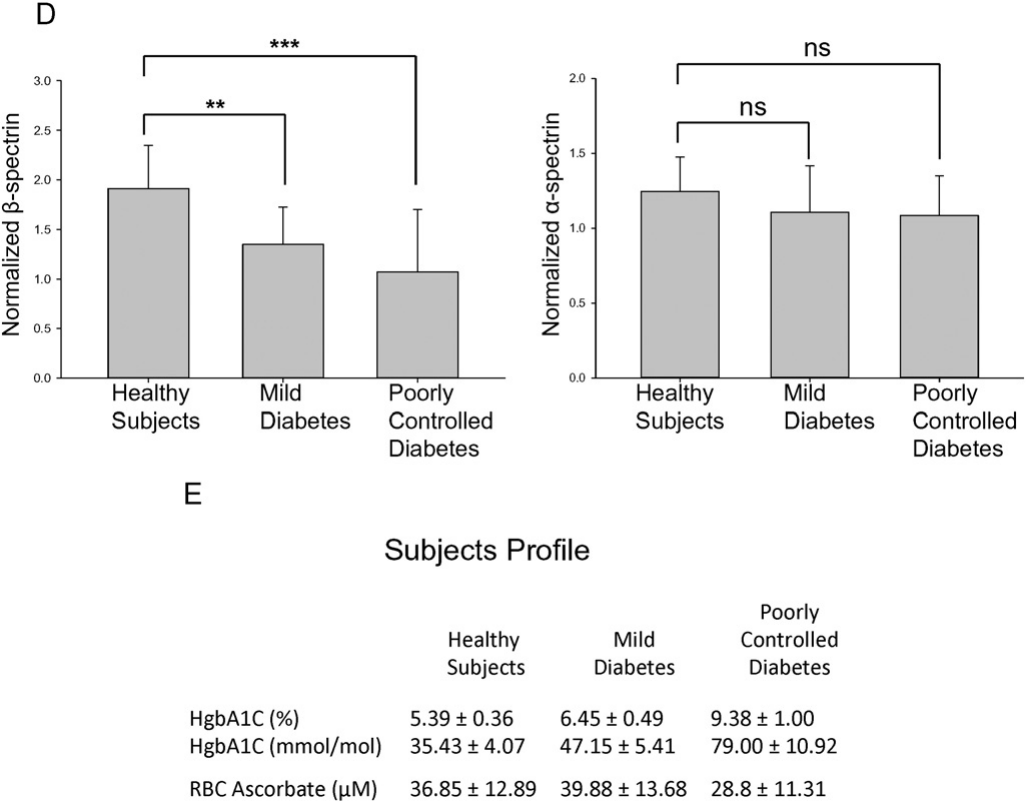
RBC ascorbate and β-spectrin in healthy and diabetic humans. A. Human RBC ascorbate concentrations as a function of plasma glucose concentrations. N = 39 and R = − 0.46. B. Osmotic fragility in human RBCs as a function of RBC ascorbate concentrations. Symbols as follows: (), healthy subjects hemoglobin A1C 4.5–5.6; (), diabetic subjects hemoglobin A1C (HgbA1C) 5.7–7.7; (), diabetic subjects hemoglobin A1C 7.8–10.6. Statistics for the line describing all data: N = 42 and R = − 0.41. Differences between () healthy subjects vs () diabetic subjects were statistically significant, p < 0.05. C, D, E. Human RBC α-spectrin, β-spectrin, and β-actin in healthy subjects (14); subjects with mild diabetes (13); and subjects with poorly controlled diabetes (13). Normalized values are in D and subject profiles in E. Subjects were randomly selected based on hemoglobin A1C and sample availability.

Due to these distinct *GLUT* transporter differences on RBCs from mice and humans, we characterized human RBCs from healthy and diabetic subjects with respect to ascorbate, osmotic fragility, and β-spectrin. Ascorbate in RBCs from these subjects was inversely correlated with fasting glucose (Fig. 7A, supplementary Fig. 7); and osmotic fragility and severity of diabetes (Fig. 7B). For osmotic fragility, p < 0.05 for healthy subjects vs. poorly controlled diabetic subjects (hemoglobin A1C > 7.8). The clinical data describing osmotic fragility in relation to ascorbate concentration are consistent with mouse findings (Fig. 2). Similarly, the clinical data indicating that hyperglycemia is associated with lower RBC ascorbate concentrations are consistent with mouse findings (Fig. 5). Compared to healthy subjects, β-spectrin progressively declined in subjects with mild and poorly controlled diabetes, while α-spectrin was unchanged (Fig. 7C, D, E). RBC ascorbate values are shown for these subject groups in Fig. 7E. A linear relationship existed between plasma and RBC ascorbate with hyperglycemia as well as with euglycemia (supplementary Fig. 7). With data displayed in this fashion, it was recapitulated that RBC ascorbate was significantly lower with hyperglycemia compared to euglycemia (supplementary Fig. 7). How hyperglycemia dynamically affects RBC ascorbate will be best determined in the same diabetic patients with and without hyperglycemia in future clinical experiments.

## 4 Discussion

The data presented here provide original links between red blood cell osmotic fragility, vitamin C, and diabetes. Hemolysis was observed when whole blood was centrifuged from mice with low vitamin C plasma and RBC concentrations. Similar low vitamin C concentrations were found in some human subjects from random sampling, and were consistently produced in mice unable to synthesize vitamin C. Mouse RBC hemolysis induced by low RBC ascorbate concentrations was quantitated by osmotic fragility, and reversed when mice were repleted with vitamin C. Mouse RBCs with increased fragility induced by low RBC ascorbate were spherocyte-like, with decreased β-spectrin. With repletion of RBC ascorbate, osmotic fragility and spherocyte appearance reversed, and β-spectrin was restored. Entry of vitamin C into RBCs was only via the oxidized form dehydroascorbic acid: ascorbic acid itself was not transported. Because dehydroascorbic acid and glucose have similar affinities for facilitated glucose transporters *in vitro*, our findings suggested an innovative connection between dehydroascorbic acid and diabetes. The putative link was strengthened by experiments using mouse and human RBCs. Advantages of studies here with dehydroascorbic acid are that dehydroascorbic acid was synthesized *de novo* and its disappearance accounted for. Dehydroascorbic acid transport, performed for the first time approximating physiologic concentrations, was progressively inhibited by glucose concentrations found in diabetes (Fig. 4A and B). Glucose concentrations *in vivo*, from diabetic mice and WT controls, were inversely associated with RBC ascorbate concentrations. Despite differences in *GLUT* identities in mouse versus human RBCs, human RBC ascorbate concentrations were again inversely related to plasma glucose, osmotic fragility, and severity of diabetes. β-spectrin progressively declined in RBCs as diabetes worsened.

**Fig. 8:**
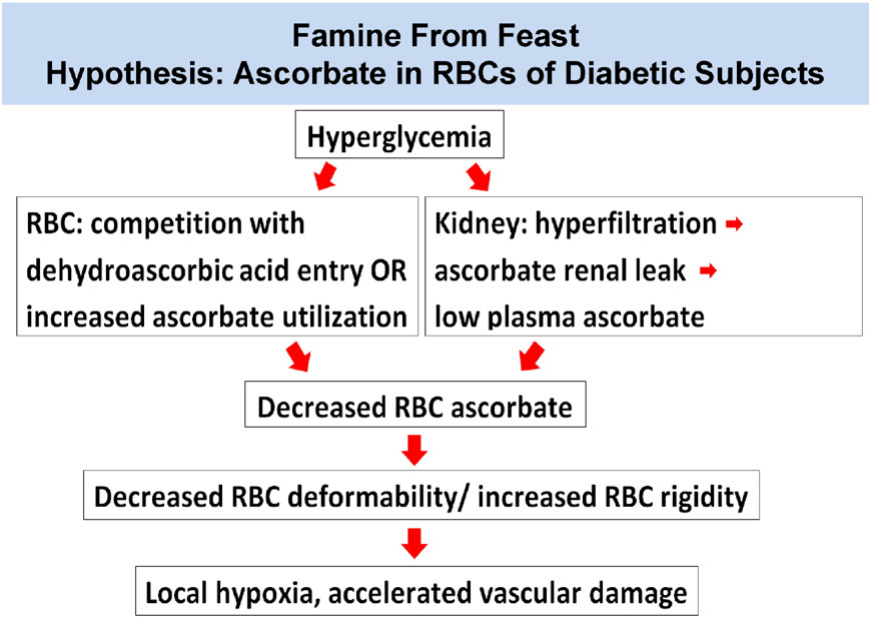
Famine From Feast Hypothesis.

Considered together, these unique findings support several parts of an original overarching hypothesis, famine from feast (Fig. 8), and provide innovative mechanism links between vitamin C and diabetes. In essence, diabetes pathophysiology might produce ascorbate deficiency in RBCs, with potential vascular pathophysiologic consequences. Increased RBC rigidity from low ascorbate would cause microvascular, or local, hypoxia by slowing RBC capillary flow, thereby decreasing oxygen delivery per unit time. The exciting therapeutic aspect is that local deficiency might be reversible with oral ascorbic acid or dehydroascorbic acid or both, or intravenous ascorbic acid (Lamas et al., 2013).

In both mice and humans, data indicated that ascorbate concentrations in RBCs were inversely related to hyperglycemia. Competition *in vivo* between DHA and glucose is an attractive explanation, but there are multiple paths that could lead to low RBC ascorbate in diabetes. For RBCs, diabetes in humans could result in: downregulation of *GLUT1* transporter expression; change in *GLUT* transporter type expressed; reduced avidity of *GLUT 1* transporters for DHA, i.e. from oxidative damage (Nishikawa et al., 2000, Yu et al., 2006, Wang et al., 2012); competition between DHA and elevated glucose concentrations; accelerated ascorbate utilization coupled to sorbitol formation within RBCs or exogenous oxidant generation from hyperglycemia (Nishikawa et al., 2000, Yu et al., 2006, Wang et al., 2012); or altered vitamin C – vitamin E recycling. Beyond RBCs, plasma ascorbate may be lower in diabetes due to: less ascorbate intake; decreased ascorbate intestinal absorption; increased glomerular filtration rate (GFR) or *SVCT1* impairment leading to diminished reabsorption; or increased ascorbate utilization. If for any of these reasons plasma vitamin C concentrations were lower, less dehydroascorbic acid would then be available for RBC entry.

Anemia is common in diabetes, with multiple causes (Thomas et al., 2003, Thomas et al., 2006, Craig et al., 2005). Low RBC ascorbate might contribute to anemia, possibly via low grade chronic hemolysis produced by RBC rigidity. Future studies of diabetic subjects will include measurement of cell-free hemoglobin and reticulocyte counts in relation to ascorbate. Future studies will also address whether increased RBC osmotic fragility caused by low ascorbate has consequences to hemoglobin function.

Consistent with the famine from feast hypothesis (Fig. 8), it has been reported that low vitamin C plasma concentrations occur in diabetes (Will and Byers, 1996, Chen et al., 2006). However, prior associations between vitamin C and diabetes did not account mechanistically for vascular complications, other than by generalized oxidative damage. Except for the dataset presented here, there has been no recognition that humans with diabetes have lower than expected vitamin C concentrations in their RBCs. Unfortunately, most prior existing datasets for plasma vitamin C concentrations in diabetic subjects are confounded by assay artifacts; by prior inabilities to measure RBC ascorbate; and by inabilities to measure dehydroascorbic acid directly in plasma or RBCs (Will and Byers, 1996, Li et al., 2012, Dhariwal et al., 1991, Lykkesfeldt, 2000). Surprisingly, modern hematology textbooks do not even describe that there is vitamin C in RBCs (Kaushansky et al., 2010, Greer et al., 2013). To our knowledge, the data here displaying RBC vs plasma vitamin C concentrations are the most comprehensive available to date in healthy and diabetic humans.

Previous investigators found no link between dehydroascorbic acid transport and glucose in mouse and human RBCs (Montel-Hagen et al., 2008a), but the findings may have been due to use of radiolabel without any direct ascorbate measurements; dehydroascorbic acid degradation (Rumsey et al., 1997); flawed kinetics assumptions (Rumsey et al., 1997, Carruthers and Naftalin, 2009); metabolism of glucose and the analog 2-deoxyglucose (Carruthers et al., 2009); and lack of accounting for *GLUT* transactivation (Cloherty et al., 1996). Based on findings on human RBCs, high co-expression of *GLUT 1* with stomatin was interpreted to enhance DHA transport but negatively modulate glucose uptake specifically in human RBCs (Montel-Hagen et al., 2008b). Recent data using RBCs and inside out vesicles do not support a role for a stomatin-regulated pool of *GLUT 1* that preferentially transports DHA rather than glucose. Instead, DHA and glucose were transported similarly by *GLUT 1* (Rumsey et al., 1997, Sage and Carruthers, 2014). The presence of *Glut 4* in mouse RBCs was previously reported (Montel-Hagen et al., 2008b), and confirmed in the results here. While *Glut*-dependent DHA uptake was concluded to be absent from mature murine RBCs (Montel-Hagen et al., 2008b), the findings in the present manuscript suggest this conclusion is incorrect.

In the current experiments we have not assessed dehydroascorbic acid transport activity of all 14 identified *GLUTs* (Mueckler and Thorens, 2013). In previous experiments, *Xenopus* oocytes were microinjected with cRNAs for *GLUTs 1–12* to assess DHA transport activity. *GLUTs 1* and *3* were at least 10 fold more active than other transporters (Rumsey et al., 1997, Rumsey et al., 2000, Corpe et al., 2013). Data here are consistent with these reports. There are no data available describing DHA transport by *GLUTs 13* and *14*. *GLUT 13* is expressed predominantly in brain, and transports myo-inositol but not glucose. Although *GLUT 14* may have arisen as a gene duplication of *GLUT 3*, there is no rodent homolog, and *GLUT 14* is expressed mainly in testis. Relying on identities of *GLUTs* in human RBCs, *GLUT* transport activity for DHA, and known distribution of *GLUTs 1–14*, we and others conclude that in human RBCs DHA is transported by *GLUT 1* (Montel-Hagen et al., 2008b, Sage and Carruthers, 2014). Based on similar reasons, we conclude that mouse RBCs utilize *Glut 3* and *Glut 4* for DHA transport, but it is unclear which transporter predominates.

DHA uptake is unlikely to be due to simple diffusion for several reasons (see Fig. 4, Fig. 6). Unlabeled 3-O-methylglucose inhibited [^3^H]3-O-methylglucose uptake and [^14^C]DHA uptake similarly. Likewise, cytochalasin B inhibited [^14^C]DHA uptake and [^3^H]3-O-methylglucose uptake in a comparable manner. In *Xenopus* oocytes, DHA transport occurred only when *GLUTs* were injected. There was no uptake with sham injection. Together, these data support the conclusion that DHA is transported on glucose transporters. While DHA diffusion cannot be excluded in mammalian cells, glucose as a similar molecule requires a membrane transporter and is transported approximately 10^4^ faster than diffusion (Elbrink and Bihler, 1975, Mueckler et al., 1985).

We show here that RBC β-spectrin decreased when RBC ascorbate concentrations were low. With ascorbate repletion, decreased β-spectrin was restored over as short a time as three days. The average life span of mouse RBCs is ~ 40 days (Van Putten, 1958). Thus, β-spectrin recovery is not due to appearance of new RBCs. We speculate that changes in β-spectrin may be due to reversible oxidative modification that is specific for ascorbate as an electron donor in RBCs. Although the full β-spectrin crystal structure is not available, no disulfide bonds were detected in the available crystal structure, suggesting that potential oxidative modification(s) occur at other sites (Ipsaro et al., 2010). Future experiments will explore these possibilities. Hemoglobin and spectrin can form complexes that may be facilitated by oxidation. In one report, complexes formed upon hydrogen peroxide treatment in vitro (Snyder et al., 1985), but only 0–5.9% of spectrin-hemoglobin was cross-linked by treating with 45–810 μM H_2_O_2_. In another report, there were only trace amounts of a spectrin-hemoglobin complex which was detected on Western blot using antibody against hemoglobin (Fortier et al., 1988). In our studies, we did not observe any shifted spectrin bands using either antibody against both α and β spectrins or specific β-spectrin antibody, suggesting that a spectrin-hemoglobin complex is below detection limits. The existence of other ascorbate- specific effects is a reasonable explanation of the decreased β-spectrin levels that we observed in ascorbate depleted *Gulo*^*−*/*−*^ mice and poorly controlled diabetic subjects.

Experiments here with ascorbate in mouse RBCs provide original insights coupling RBC fragility to low ascorbate concentrations. Notably, humans have an essential requirement for ascorbate; human RBCs have far more avidity than mouse RBCs for DHA; and human and mouse RBCs have distinctly different *GLUT* transporter expression. These species differences matter, and therefore human rather than rodent experimentation is now the way forward. Clear clinical research possibilities exist to study RBCs in humans with diabetes in relation to ascorbate. Many possibilities are described above. Additional experimental goals in humans are to characterize RBC ascorbate concentrations dynamically in humans with diabetes, as a function of glycemia in each subject. To do so, diabetic subjects with low ascorbate can be hospitalized; their RBC deformability parameters can be measured before and during controlled hyperglycemia; after discharge, these subjects can be supplemented with ascorbate to increase their RBC ascorbate concentrations; post supplementation, subjects can be re-hospitalized to again measure RBC properties with controlled hyperglycemia. Only after RBC ascorbate concentrations are characterized dynamically in diabetic subjects can longer term goals be approached. These include determination of oxygen delivery to a relevant appropriate microvascular circulation, and/or clinical outcomes (i.e. progression of retinopathy) as a function of RBC ascorbate concentrations. Although it will take time to address unknowns through clinical experimentation, the experimental outcomes have the possibility to substantially impact clinical practice, and initial studies have begun (NCT02107976 and NCT00071526, clinical trials.gov).

## Funding and Role of Funder

This work was supported by the Intramural Research Program of NIDDK, NIH, grant 1ZIADK053218-08.

The funding source had no role in the writing of the manuscript and the decision to submit it for publication. No pharmaceutical company or other agency was involved.

## Conflicts of Interest

No authors have any conflicts of interest.

## Author Contributions

HT, HL, Yu W., MN, JL, and ML planned overall concepts.

HT, HL, Yu W., MN, Yaohui W., JL, and ML designed experiments.

HT, HL, Yu W., MN, JL, Yaohui W performed experiments.

HT, HL, Yu W., MN, Yaohui W., JL, and ML interpreted data.

HT, Yu W., MN, and ML wrote the paper.

## References

[bb0005] Ali S.M., Chakraborty S.K. (1989). Role of Plasma Ascorbate in Diabetic Microangiopathy. Bangladesh Med. Res. Counc. Bull..

[bb0010] American Diabetes Association: Position Statement (2014). Diagnosis and Classification of Diabetes Mellitus. Diabetes Care.

[bb0015] American Diabetes Association: Position Statement (2015). Classification and Diagnosis of Diabetes. Diabetes Care.

[bb0020] Beckman J.A., Creager M.A., Libby P. (2002). Diabetes and Atherosclerosis: Epidemiology, Pathophysiology, and Management. J. Am. Med. Assoc..

[bb0025] Bergsten P., Amitai G., Kehrl J., Dhariwal K.R., Klein H.G., Levine M. (1990). Millimolar Concentrations of Ascorbic Acid in Purified Human Mononuclear Leukocytes. Depletion and reaccumulation. J. Biol. Chem..

[bb0030] Bianchi J., Rose R.C. (1986). Glucose-Independent Transport of Dehydroascorbic Acid in Human Erythrocytes. Proc. Soc. Exp. Biol. Med..

[bb0035] Brown C.D., Ghali H.S., Zhao Z., Thomas L.L., Friedman E.A. (2005). Association of Reduced red Blood Cell Deformability and Diabetic Nephropathy. Kidney Int..

[bb0040] Buys A.V., Van Rooy M.J., Soma P., Van P.D., Lipinski B., Pretorius E. (2013). Changes in red blood cell membrane structure in type 2 diabetes: a scanning electron and atomic force microscopy study. Cardiovasc. Diabetol..

[bb0045] Carruthers A., Naftalin R.J. (2009). Altered GLUT1 Substrate Selectivity in Human Erythropoiesis?. Cell.

[bb0050] Carruthers A., DeZutter J., Ganguly A., Devaskar S.U. (2009). Will the Original Glucose Transporter Isoform Please Stand up!. Am. J. Physiol. Endocrinol. Metab..

[bb0055] Chazan J.A., Mistilis S.P. (1963). The Pathophysiology of Scurvy. A report of seven cases. Am. J. Med..

[bb0060] Chen H., Karne R.J., Hall G., Campia U., Panza J.A., Cannon R.O., Wang Y., Katz A., Levine M., Quon M.J. (2006). High-Dose Oral Vitamin C Partially Replenishes Vitamin C Levels in Patients with Type 2 Diabetes and low Vitamin C Levels but Does not Improve Endothelial Dysfunction or Insulin Resistance. Am. J. Physiol. Heart Circ. Physiol..

[bb0065] Clark M.R., Mohandas N., Shohet S.B. (1983). Osmotic Gradient Ektacytometry: Comprehensive Characterization of red Cell Volume and Surface Maintenance. Blood.

[bb0070] Cloherty E.K., Heard K.S., Carruthers A. (1996). Human Erythrocyte Sugar Transport is Incompatible with Available Carrier Models. Biochemistry.

[bb0075] Concha I.I., Velasquez F.V., Martinez J.M., Angulo C., Droppelmann A., Reyes A.M., Slebe J.C., Vera J.C., Golde D.W. (1997). Human Erythrocytes Express GLUT5 and Transport Fructose. Blood.

[bb0080] Corpe C.P., Eck P., Wang J., Al-Hasani H., Levine M. (2013). Intestinal Dehydroascorbic Acid (DHA) Transport Mediated by the Facilitative Sugar Transporters, GLUT2 and GLUT8. J. Biol. Chem..

[bb0085] Cox E.V. (1968). The Anemia of Scurvy. Vitam. Horm..

[bb0090] Craig K.J., Williams J.D., Riley S.G., Smith H., Owens D.R., Worthing D., Cavill I., Phillips A.O. (2005). Anemia and Diabetes in the Absence of Nephropathy. Diabetes Care.

[bb0095] Cubbon R.M., Mercer B.N., Sengupta A., Kearney M.T. (2013). Importance of insulin resistance to vascular repair and regeneration.. Free Radic. Biol. Med..

[bb0100] Cunningham J.J. (1998). The Glucose/Insulin System and Vitamin C: Implications in Insulin-Dependent Diabetes Mellitus. J. Am. Coll. Nutr..

[bb0105] Dhariwal K.R., Hartzell W.O., Levine M. (1991). Ascorbic Acid and Dehydroascorbic Acid Measurements in Human Plasma and Serum. Am. J. Clin. Nutr..

[bb0110] Diamantopoulos E.J., Kittas C., Charitos D., Grigoriadou M., Ifanti G., Raptis S.A. (2004). Impaired Erythrocyte Deformability Precedes Vascular Changes in Experimental Diabetes Mellitus. Horm. Metab. Res..

[bb0115] Diez-Silva M., Dao M., Han J., Lim C.T., Suresh S. (2010). Shape and Biomechanical Characteristics of Human Red Blood Cells in Health and Disease. MRS Bull..

[bb0120] Elbrink J., Bihler I. (1975). Membrane transport: its relation to cellular metabolic rates. Science.

[bb0125] Fioretto P., Dodson P.M., Ziegler D., Rosenson R.S. (2010). Residual Microvascular Risk in Diabetes: Unmet Needs and Future Directions. Nat. Rev. Endocrinol..

[bb0130] Fortier N., Snyder L.M., Garver F., Kiefer C., McKenney J., Mohandas N. (1988). The Relationship Between in Vivo Generated Hemoglobin Skeletal Protein Complex and Increased red Cell Membrane Rigidity. Blood.

[bb0135] Freckmann G., Schmid C., Baumstark A., Pleus S., Link M., Haug C. (2012). System Accuracy Evaluation of 43 Blood Glucose Monitoring Systems for Self-Monitoring of Blood Glucose According to DIN EN ISO 15197. J. Diabetes Sci. Technol..

[bb0140] Greer J.P., Arber D.A., Glader B., List A.F., Means R.F., Paraskevas F., Rodgers G.M. (2013). Wintrobe's Clinical Hematology.

[bb0145] Hart H.C., Ploem J.E., Panders J.T., Verloop M.C. (1964). Haemorrhagic Diathesis and Anaemia in Scurvy. Report on Three Cases. Acta Med. Scand..

[bb0150] Hughes R.E., Maton S.C. (1968). The Passage of Vitamin C Across the Erythrocyte Membrane. Br. J. Haematol..

[bb0155] Ipsaro J.J., Harper S.L., Messick T.E., Marmorstein R., Mondragon A., Speicher D.W. (2010). Crystal Structure and Functional Interpretation of the Erythrocyte Spectrin Tetramerization Domain Complex. Blood.

[bb0160] Kageyama K., Onoyama Y., Kogawa H., Goto E., Tanabe K. (1989). The Maximum and Minimum Water Content and Cell Volume of Human Erythrocytes in Vitro. Biophys. Chem..

[bb0165] Kamada T., McMillan D.E., Yamashita T., Otsuji S. (1992). Lowered Membrane Fluidity of Younger Erythrocytes in Diabetes. Diabetes Res. Clin. Pract..

[bb0170] Kaushansky K., Lichtman M.A., Beutler E., Kipps T.J., Seligsohn U., Prchal J.T. (2010). Williams Hematology.

[bb0175] Keymel S., Heiss C., Kleinbongard P., Kelm M., Lauer T. (2011). Impaired red Blood Cell Deformability in Patients with Coronary Artery Disease and Diabetes Mellitus. Horm. Metab. Res..

[bb0180] Kung C.M., Tseng Z.L., Wang H.L. (2009). Erythrocyte Fragility Increases with Level of Glycosylated Hemoglobin in Type 2 Diabetic Patients. Clin. Hemorheol. Microcirc..

[bb0185] Lamas G.A., Goertz C., Boineau R., Mark D.B., Rozema T., Nahin R.L., Lindblad L., Lewis E.F., Drisko J., Lee K.L. (2013). Effect of Disodium EDTA Chelation Regimen on Cardiovascular Events in Patients with Previous Myocardial Infarction: The TACT Randomized Trial. J. Am. Med. Assoc..

[bb0190] Levine M., Conry-Cantilena C., Wang Y., Welch R.W., Washko P.W., Dhariwal K.R., Park J.B., Lazarev A., Graumlich J., King J., Cantilena L.R. (1996). Vitamin C Pharmacokinetics in Healthy Volunteers: Evidence for a Recommended Dietary Allowance. Proc. Natl. Acad. Sci. U. S. A..

[bb0195] Levine M., Wang Y., Padayatty S.J., Morrow J. (2001). A new Recommended Dietary Allowance of Vitamin C for Healthy Young Women. Proc. Natl. Acad. Sci. U. S. A..

[bb0200] Li H., Tu H., Wang Y., Levine M. (2012). Vitamin C in Mouse and Human red Blood Cells: An HPLC Assay. Anal. Biochem..

[bb0205] Lindsay R.M., Jamieson N.S., Walker S.A., McGuigan C.C., Smith W., Baird J.D. (1998). Tissue Ascorbic Acid and Polyol Pathway Metabolism in Experimental Diabetes. Diabetologia.

[bb0210] Lykkesfeldt J. (2000). Determination of Ascorbic Acid and Dehydroascorbic Acid in Biological Samples by High-Performance Liquid Chromatography Using Subtraction Methods: Reliable Reduction with Tris[2-Carboxyethyl]Phosphine Hydrochloride. Anal. Biochem..

[bb0215] Maeda N., Hagihara H., Nakata Y., Hiller S., Wilder J., Reddick R. (2000). Aortic Wall Damage in Mice Unable to Synthesize Ascorbic Acid. Proc. Natl. Acad. Sci. U. S. A..

[bb0220] Mann G.V., Newton P. (1975). The Membrane Transport of Ascorbic Acid. Ann. N. Y. Acad. Sci..

[bb0225] May J.M., Qu Z., Morrow J.D. (2001). Mechanisms of Ascorbic Acid Recycling in Human Erythrocytes. Biochim. Biophys. Acta.

[bb0230] May J.M., Qu Z.C., Qiao H., Koury M.J. (2007). Maturational Loss of the Vitamin C Transporter in Erythrocytes. Biochem. Biophys. Res. Commun..

[bb0235] McMillan D.E., Utterback N.G., La P.J. (1978). Reduced Erythrocyte Deformability in Diabetes. Diabetes.

[bb0240] Mendiratta S., Qu Z.C., May J.M. (1998). Erythrocyte Ascorbate Recycling: Antioxidant Effects in Blood. Free Radic. Biol. Med..

[bb0245] Moitra J., Mason M.M., Olive M., Krylov D., Gavrilova O., Marcus-Samuels B., Feigenbaum L., Lee E., Aoyama T., Eckhaus M., Reitman M.L., Vinson C. (1998). Life Without White fat: A Transgenic Mouse. Genes Dev..

[bb0250] Montel-Hagen A., Blanc L., Boyer-Clavel M., Jacquet C., Vidal M., Sitbon M., Taylor N. (2008). The Glut1 and Glut4 Glucose Transporters are Differentially Expressed During Perinatal and Postnatal Erythropoiesis. J. Clin. Invest..

[bb0255] Montel-Hagen A., Kinet S., Manel N., Mongellaz C., Prohaska R., Battini J.L., Delaunay J., Sitbon M., Taylor N. (2008). Erythrocyte Glut1 Triggers Dehydroascorbic Acid Uptake in Mammals Unable to Synthesize Vitamin C. Cell.

[bb0260] Mueckler M., Thorens B. (2013). The SLC2 (GLUT) Family of Membrane Transporters. Mol. Asp. Med..

[bb0265] Mueckler M., Caruso C., Baldwin S.A., Panico M., Blench I., Morris H.R., Allard W.J., Lienhard G.E., Lodish H.F. (1985). Sequence and Structure of a Human Glucose Transporter. Science.

[bb0270] Nishikawa T., Edelstein D., Du X.L., Yamagishi S., Matsumura T., Kaneda Y., Yorek M.A., Beebe D., Oates P.J., Hammes H.P., Giardino I., Brownlee M. (2000). Normalizing Mitochondrial Superoxide Production Blocks Three Pathways of Hyperglycaemic Damage. Nature.

[bb0275] Padayatty S.J., Katz A., Wang Y., Eck P., Kwon O., Lee J.H., Chen S., Corpe C., Dutta A., Dutta S.K., Levine M. (2003). Vitamin C as an Antioxidant: Evaluation of its Role in Disease Prevention. J. Am. Coll. Nutr..

[bb0280] Parpart A.K., Lorenz P.B., Parpart E.R., Gregg J.R., Chase A.M. (1947). The Osmotic Resistance (Fragility) of Human red Cells. J. Clin. Invest..

[bb0285] Parthasarathi K., Lipowsky H.H. (1999). Capillary Recruitment in Response to Tissue Hypoxia and its Dependence on red Blood Cell Deformability. Am. J. Physiol..

[bb0290] Peterson C.M., Jones R.L., Koenig R.J., Melvin E.T., Lehrman M.L. (1977). Reversible Hematologic Sequelae of Diabetes Mellitus. Ann. Intern. Med..

[bb0295] Rumsey S.C., Daruwala R., Al-Hasani H., Zarnowski M.J., Simpson I.A., Levine M. (2000). Dehydroascorbic Acid Transport by GLUT4 in Xenopus Oocytes and Isolated rat Adipocytes. J. Biol. Chem..

[bb0300] Rumsey S.C., Kwon O., Xu G.W., Burant C.F., Simpson I., Levine M. (1997). Glucose Transporter Isoforms GLUT1 and GLUT3 Transport Dehydroascorbic Acid. J. Biol. Chem..

[bb0305] Sacks D.B., Burtis B.A., Ashwood E.R., Bruns D.E., Sawyer B.G. (2008). Carbohydrates. Tietze Fundamentals of Clinical Chemistry.

[bb0310] Sage J.M., Carruthers A. (2014). Human Erythrocytes Transport Dehydroascorbic Acid and Sugars Using the Same Transporter Complex. Am. J. Physiol. Cell Physiol..

[bb0315] Shin S., Ku Y., Babu N., Singh M. (2007). Erythrocyte Deformability and its Variation in Diabetes Mellitus. Indian J. Exp. Biol..

[bb0320] Simchon S., Jan K.M., Chien S. (1987). Influence of Reduced red Cell Deformability on Regional Blood Flow. Am. J. Physiol..

[bb0325] Sinclair A.J., Girling A.J., Gray L., Le Guen C., Lunec J., Barnett A.H. (1991). Disturbed Handling of Ascorbic Acid in Diabetic Patients with and Without Microangiopathy During High Dose Ascorbate Supplementation. Diabetologia.

[bb0330] Snyder L.M., Fortier N.L., Trainor J., Jacobs J., Leb L., Lubin B., Chiu D., Shohet S., Mohandas N. (1985). Effect of Hydrogen Peroxide Exposure on Normal Human Erythrocyte Deformability, Morphology, Surface Characteristics, and Spectrin-Hemoglobin Cross-Linking. J. Clin. Invest..

[bb0335] Som S., Basu S., Mukherjee D., Deb S., Choudhury P.R., Mukherjee S., Chatterjee S.N., Chatterjee I.B. (1981). Ascorbic Acid Metabolism in Diabetes Mellitus. Metabolism.

[bb0340] Sotiriou S., Gispert S., Cheng J., Wang Y., Chen A., Hoogstraten-Miller S., Miller G.F., Kwon O., Levine M., Guttentag S.H., Nussbaum R.L. (2002). Ascorbic-Acid Transporter Slc23a1 is Essential for Vitamin C Transport into the Brain and for Perinatal Survival. Nat. Med..

[bb0345] Steck T.L., Kant J.A. (1974). Preparation of Impermeable Ghosts and Inside-out Vesicles from Human Erythrocyte Membranes. Methods Enzymol..

[bb0350] Stein W.D., Stein W.D. (1986). Facilitated Diffusion: the simple carrier. Transport and Diffusion Across Cell Membranes.

[bb0355] Tanabe K., Kageyama K., Takada S. (1986). An Increase in Water Content of Mouse Erythrocytes Infected with Plasmodium Yoelii. Trans. R. Soc. Trop. Med. Hyg..

[bb0360] Thomas M.C., MacIsaac R.J., Tsalamandris C., Power D., Jerums G. (2003). Unrecognized Anemia in Patients with Diabetes: A Cross-Sectional Survey. Diabetes Care.

[bb0365] Thomas M.C., Tsalamandris C., MacIsaac R.J., Jerums G. (2006). The Epidemiology of Hemoglobin Levels in Patients with Type 2 Diabetes. Am. J. Kidney Dis..

[bb0370] Van Putten L.M. (1958). The Life Span of red Cells in the rat and the Mouse as Determined by Labeling with DFP32 in Vivo. Blood.

[bb0375] Vera J.C., Rivas C.I., Fischbarg J., Golde D.W. (1993). Mammalian Facilitative Hexose Transporters Mediate the Transport of Dehydroascorbic Acid. Nature.

[bb0380] Virtue M.A., Furne J.K., Nuttall F.Q., Levitt M.D. (2004). Relationship Between GHb Concentration and Erythrocyte Survival Determined from Breath Carbon Monoxide Concentration. Diabetes Care.

[bb0385] Wang W., Wang Y., Long J., Wang J., Haudek S.B., Overbeek P., Chang B.H., Schumacker P.T., Danesh F.R. (2012). Mitochondrial Fission Triggered by Hyperglycemia is Mediated by ROCK1 Activation in Podocytes and Endothelial Cells. Cell Metab..

[bb0390] Will J.C., Byers T. (1996). Does Diabetes Mellitus Increase the Requirement for Vitamin C?. Nutr. Rev..

[bb0395] Wong W.T., Wong S.L., Tian X.Y., Huang Y. (2010). Endothelial Dysfunction: The Common Consequence in Diabetes and Hypertension. J. Cardiovasc. Pharmacol..

[bb0400] Yau T.W., Kuchel R.P., Koh J.M., Szekely D., Mirtschin P.J., Kuchel P.W. (2012). Cytoskeletal Rearrangements in Human red Blood Cells Induced by Snake Venoms: Light Microscopy of Shapes and NMR Studies of Membrane Function. Cell Biol. Int..

[bb0405] Yu T., Robotham J.L., Yoon Y. (2006). Increased Production of Reactive Oxygen Species in Hyperglycemic Conditions Requires Dynamic Change of Mitochondrial Morphology. Proc. Natl. Acad. Sci. U. S. A..

