# Supplementary material for: Low Red Blood Cell Vitamin C Concentrations Induce Red Blood Cell Fragility: A Link to Diabetes Via Glucose, Glucose Transporters, and Dehydroascorbic Acid
Source: eBioMedicine. 2015 Oct 3;2(11):1735–50. doi: 10.1016/j.ebiom.2015.09.049 (PMC4740302; doi:10.1016/j.ebiom.2015.09.049)
Supplement: Supplementary file 1 — Supplementary material [file mmc1.pdf]

SUPPLEMENTARY METHODS

Mice and blood samples from mice

Homozygous *Gulo*$^{-/-}$ mice were bred from obtained heterozygote mice (B6.129P2-GulotmlUmc/mmced, backcrossed to C57BL/6 for 10 generations). *Gulo*$^{-/-}$ mice were confirmed by RT-PCR and vitamin C deprivation to lack L-gulono-γ-lactone oxidase and the ability to synthesize vitamin C, respectively (Maeda et al., 2000). AZIP mice have virtually no white fat; have diabetes with hyperglycemia and hyperinsulinemia; and do not develop ketoacidosis (Moitra et al., 1998).

For experiments to determine plasma and/or RBC ascorbate, approximately 60-80 µL of blood was obtained by mandibular puncture and flowed into capillary tubes by capillary action (Li et al., 2012). For experiments in which osmotic fragility was determined, sample volume was a maximum of 150 µL. For mouse experiments requiring more than 150 µL of blood, mice were anaesthetized with isofluorane and approximately 400 µl whole blood was obtained by orbital puncture prior to sacrifice by cervical dislocation.

For experiments describing RBC ascorbate in relation to plasma ascorbate and osmotic fragility (fig 2B,2C): 5 *Gulo*$^{-/-}$ male mice were supplemented with 1g/L ascorbate in drinking water, and sampled every other week; 10 *Gulo*$^{-/-}$ male mice were unsupplemented for 14 weeks, followed by gavage of 0.2mg ascorbate in 100 µL water at 14 and 17 weeks. Gavage was performed to maintain mice at low ascorbate values while preventing demise from scurvy. For unsupplemented *Gulo*$^{-/-}$ mice, five were sampled every other week in rotation. Sample volume was 150 µL every other week. Depletion and supplementation doses times are described in figure legends.

Erythrocyte osmotic fragility

Osmotic fragility was based on RBC lysis with varying concentrations of NaCl (Parpart et al., 1947). Mouse or human whole blood 150 µL was collected into heparinized Micro-Hematocrit capillary tubes (Fisher Scientific). One 10 µL aliquot was added into each of 11 wells on a 96-well round bottom plate. Wells contained 150 µL of either 0.90%, 0.70%, 0.65%, 0.60%, 0.55%, 0.50%, 0.45%, 0.40%, 0.35%, 0.30%, or 0.20% NaCl and one well contained 150 µL of ultrapurified water. To avoid mechanical hemolysis, every well was gently mixed by pipetting three times. Test plates were incubated for 60 minutes at room temperature (~23°C), and subsequently well contents were centrifuged at 1740 x g for 5 minutes in 4°C. The supernatant was transferred into a new 96-well flat bottom plate and hemoglobin content determined spectrophotometrically at λmax = 540 nm with a µQuant™ Microplate Spectrophotometer (Bio-Tek Instruments, Inc) using supernatant from the 0.90% NaCl well as the blank. Hemolysis in each well was expressed as a percentage, taking as 100% the maximum value of absorbance of the well that contained erythrocytes suspended in distilled water. The percent of hemolysis was calculated by: % Hemolysis = (O.D. of test well - O.D. of 0.90% NaCl well) ÷ (O.D. of dH_2O well - O.D. of 0.90% NaCl well).

Xenopus laevis oocyte transport assay

Xenopus laevis oocytes were isolated and injected with cRNAs as described (Corpe et al., 2013). Individual oocytes (stages V and VI) were isolated from connective tissue and vasculature, transferred to calcium-containing media (OR-2 with 1 mM $CaCl_2$), and maintained at 18-20 °C until injection with cRNA. Oocytes were injected utilizing a Nanoject II injector (Drummond Scientific). Injection volumes were 36.8 nL, and cRNA concentrations were

1 ng/nL. Sham-injected oocytes were injected with 36.8 nL of water. Post-injected oocytes were incubated at 18-20 °C in OR-2 containing 1mM $CaCl_2$ and 1 mM pyruvate with daily media changes until uptake experiments were performed. Three days post-injection, oocytes were equilibrated at room temperature in media. To begin uptake experiments, freshly prepared [^{14}C] DHA (0.6-5.5 μCi/ml) was added at the indicated concentrations for 10 min. After incubation at room temperature, oocytes were washed four times with ice-cold phosphate-buffered saline. Individual oocytes per replicate were solubilized with 10% SDS, and internalized radioactivity was quantified by scintillation spectrometry as pmol/oocyte. Each data point represents the mean ± SD of 10 oocytes.

Acute in vitro supplementation of RBCs for measurement of β-spectrin

Freshly isolated RBCs from ascorbate supplemented or unsupplemented male *Gulo*$^{-/-}$ mice were washed three times using cold PBS, and resuspended to a final 10% hematocrit in PBS. Freshly prepared ascorbate or dehydroascorbic acid in PBS was added to mouse RBCs to make final concentrations of 50 μM. RBCs were incubated at 37°C for 2 min to allow uptake. RBCs were then washed twice using PBS. One aliquot was lysed using modified RIPA lysis buffer for western blot analysis, and another aliquot was lysed with water for ascorbic acid analysis.

Western Blot

For sample preparation, 6 μL of red cell lysate in modified RIPA buffer was reduced by mixing with 2 μL of NuPAGE LDS sample buffer (4X, containing 5% 2-mercaptoethanol) and boiled for 3 min before loading into each lane of western blot gel.

Western blot samples were separated in pre-casted NuPAGE 4-12% Bis-Tris protein gels in Xcell II Mini-Cell system (Life technologies, Carlsbad, CA) at 160 V for 2h. Samples were then transferred onto nitrocellulose membranes using Novex Tris-Glycine transfer buffer (Life technologies) in Mini Trans-Blot Cell (Bio-Rad, Hercules, CA) at 300 mA for 4h. The resulting membranes were incubated with corresponding antibodies. For *Glut 1* Western blots: NIH/3T3 cell (mouse embryonic fibroblast) lysate was added as positive control for mouse RBC. For *GLUT 2* Western blots: Caco-2 cell (human colon cancer cell) lysate was added as positive control for human RBCs, and mouse intestine lysate was added as positive control for mouse RBCs. For *GLUT 3* Western blots: U87-MG cell (human glioblastoma cell) lysate was added for human RBCs, and mouse brain lysate was added as positive control for mouse RBCs. For *GLUT 4* Western blots: MCF-7 cell (human breast cancer cell) was added as positive control for human RBCs, and mouse skeletal muscle and NIH/3T3 cell lysate were added as positive control for mouse RBCs.

Loading RBCs with 3-O-MG

Time course of 3-O-MG loading

50 µL of washed human RBC prepared as above were suspended in 450 µL PBS containing 5 mM or 20 mM 3-O-MG with [^{3}H]3-O-MG tracer (1µCi/mL). RBCs were incubated at 37°C for 0.5, 1, 2, 5, 10, 20, or 30 minutes. At each time point, 1 mL ice-cold stop buffer (10 µM cytochalasin B in PBS) was added and the mixture centrifuged at 200 x g for 3 min at 4° C. The supernatant was discarded, RBCs re-suspended in 1 mL stop buffer, and washed twice more at 4°C. After the third wash, 40 µL RBCs were added to 360 µL ultrapurified water and pipetted until hemolysis. The RBC lysate were transferred into a centrifugal filtration device as above,

(Amicon Ultra 10K, EMD Millipore), centrifuged at 14,000 x g for 15 min at 4°C, and 200 µL of supernatant added to 5 mL scintillation fluid (Ultima-Flo, Packard Bioscience, Meriden CT) for scintillation spectrometry analysis.

Effect of RBCs preloaded with varying 3-O-MG concentrations on subsequent 3-O-MG uptake at 4° C

Human RBCs 2 mL prepared as above were suspended in PBS buffer final volume 20 mL containing 0, 2.5, 5, 10, 15, or 20 mM 3-O-MG at 37°C. After 20 minute incubation, mixtures were centrifuged at 500 g for 5 minutes, supernatant removed, and preloaded RBCs placed on ice until use. For experiments, 50 µL preloaded RBCs were added to 450 µL ice-cold PBS buffer containing 5 mM (1µCi/mL) or 20 mM 3-O- MG(1µCi/mL) . After 1 minute incubation on ice, 1 mL ice-cold stop buffer (10µM cytochalasin B in PBS) was added and samples processed as above for scintillation spectrometry analyses.

Uptake of [^{3}H]2-deoxyglucose into human RBCs and evaluation of competition by glucose at 37°C

Human RBCs 2mL prepared as above were preincubated in PBS with 0, 5, or 25 mM glucose for 5 minutes at 37°C followed by addition of 17 µM [^{3}H]2-deoxyglucose alone for 0-60 seconds at 37°C. RBCs were also prepared as above and, without preincubation, were directly added to mixtures in PBS containing 17 µM [^{3}H]2-deoxyglucose and 0, 5, or 25 mM glucose for 0-60 seconds at 37°C. Uptake was stopped by addition of ice-cold stop buffer, and RBCs were washed and analyzed by scintillation spectrometry as described.

Glucose analyses

Mouse and human whole blood glucose were quantitated by commercial techniques. In mouse samples whole blood glucose was measured based on conversion of glucose to gluconolactone. One drop of mouse blood was added to test strips containing glucose dehydrogenase and hexacyanoferrate (III) and current generated was measured by biamperometery (Accu Chek, Roche Diagnostics). Human samples were measured by the hexokinase method. Glucose-6-phosphate dehydrogenase oxidizes glucose-6-phosphate in the presence of NADP. The rate of NADPH formation is directly proportional to glucose concentration and is measured spectophotometrically (Sacks, 2008). Values between methods used for mouse and human samples are within 10% (Freckmann et al., 2012).

SUPPLEMENTARY FIGURES

Supplementary Figure 1

Mouse RBC ascorbate concentrations, osmotic fragility, and underlying mechanism

A. RBC and plasma ascorbate concentrations in *Gulo*$^{-/-}$ mice that had not received ascorbate for weeks indicated on x axis. RBC concentrations (○) are on left y axis, plasma concentrations ● are on right y axis

B. Confocal microscopy of RBCs from ascorbate supplemented and unsupplemented *Gulo*$^{-/-}$ mice. Ascorbate supplemented *Gulo*$^{-/-}$ mice (N = 6) received ascorbate 1g/L for 12 weeks, and unsupplemented *Gulo*$^{-/-}$ mice (N = 6) had no ascorbate for 12 weeks (plasma ascorbate concentrations were 66 ± 10 and 1 ± 1 μM respectively, see Fig2G). Forty to fifty RBCs in full view orientation were randomly selected from each group for measurement of diameters.

C. Confocal microscopy of RBCs from ascorbate supplemented *Gulo*$^{-/-}$ mice and unsupplemented then resupplemented *Gulo*$^{-/-}$ mice. RBCs were obtained from continuously ascorbate supplemented *Gulo*$^{-/-}$ mice (N = 6) and unsupplemented *Gulo*$^{-/-}$ mice (N = 6)(see supplementary fig 1B above) that were then supplemented for 10 days using 2g/L ascorbate in drinking water. Plasma concentrations (fig2G) were 66 +/- 10 and 61 +/- 9 μM respectively. RBCs were prepared as in supplementary fig 1B above.

D. Hematologic properties and weights of unsupplemented and supplemented *Gulo*$^{-/-}$ mice. Mice were continuously supplemented with ascorbate 1g/L for 12 weeks (N = 5); unsupplemented for 12 weeks (N = 8); and unsupplemented followed by resupplementation for 10 days (N=6), ascorbate 2G/L to ensure rapid resupplementation. Hematologic parameters are: hematocrit; mean corpuscular volume (MCV); red blood cell distribution width (RDW); mean corpuscular hemoglobin (MCH); and mean corpuscular hemoglobin concentration (MCHC). For

these measures, 100 μL of mouse whole blood was analyzed using Hemavet model 950 (Drew Scientific, Dallas, TX), according to the manufacturer's instructions. Bottom panel second from right: reticulocyte counts, analyzed by staining with microscopy, were performed in supplemented (N=5) and unsupplemented (N = 5) *Gulo*$^{-/-}$ mice. Bottom right panel: mice weights were measured before blood was withdrawn.

E. β-Spectrin in RBC lysates from ascorbate supplemented and unsupplemented *Gulo*$^{-/-}$ female mice. *Gulo*$^{-/-}$ female mice were supplemented with ascorbate (1g/L) or unsupplemented for 7 weeks. RBC lysates obtained from whole blood were analyzed by Western blots using antibodies to β-spectrin and ankyrin-1, with β-actin as loading control. Samples from 5 supplemented and 5 unsupplemented mice are displayed. Plasma ascorbate concentrations are shown in supplementary fig 1G. Bottom panel: normalized β-spectrin values from top panel. Note that depletion times are faster in female *Gulo*$^{-/-}$ mice compared to male *Gulo*$^{-/-}$ mice.

F. . β-Spectrin in RBC lysates from ascorbate supplemented, unsupplemented, and resupplemented *Gulo*$^{-/-}$ female mice.

Top panel: *Gulo*$^{-/-}$ female mice were supplemented with ascorbate (1g/L, 4 mice, left lanes); unsupplemented for 7 weeks (4 mice, right lanes); or unsupplemented for 7 weeks followed by supplementation (2g/L) for 3 days (4 mice, middle lanes). RBC lysates obtained from whole blood were analyzed by Western blots using antibodies to β-spectrin and ankyrin-1, with β-actin as loading control.

Bottom panel: normalized β-spectrin values from top panel.

G. Plasma (left panel) and RBC (right panel) ascorbate concentrations in blood from female *Gulo*$^{-/-}$ mice in Supplementary Fig 1E,F.

H. . β-Spectrin in RBC lysates acutely supplemented *in vitro*.

Gulo$^{-/-}$ male mice were unsupplemented for 8 weeks (N = 3) or supplemented with ascorbate in drinking water 1g/L (N = 3). RBCs were obtained and incubated at 37 ^{o}C for 2 minutes with 50 µM ascorbate or dehydroascorbic acid. RBCs were analyzed for ascorbic acid content by HPLC and for β-spectrin by western blot.

Supplementary Figure 2

Measurement of extracellular dehydroascorbic acid in standards

Extracellular dehydroascorbic acid standards were prepared as 100 µM before dilution. In addition to the values in fig4 and supplementary fig2, dehydroascorbic acid concentrations were measured for all other experiments (n=7). Dehydroascorbic acid was measured by reduction to ascorbate with TCEP as described (Li et al., 2012).

Supplementary Figure 3A-B

Dehydroascorbic acid and ascorbic acid transport into human (A) and mouse (B) RBCs, extracellular concentration of 5 µM

Intended extracellular dehydroascorbic acid or ascorbate concentration was 5 µM.

A different symbol represents a different subject or mouse; for open symbols, values were determined by HPLC; for black- filled symbols, 500 µM TCEP was added during incubation, values determined by HPLC; for light grey symbols, values determined by scintillation spectrometry; for dark grey symbols, 500 µM TCEP added during incubation, values determined by scintillation spectrometry. Note that in each figure group one symbol may be open, light, and dark, indicating that values were determined using several different conditions, as above. In each figure group, the same symbol type for RBC concentration and extracellular substrate indicate corresponding (matched) samples. All symbols indicate values for $\geq$ 3 different experimental samples. For human RBC experiments, 50 µL RBCs were incubated in 0.5 mL total volume

using PBS with 5 mM glucose. For mouse RBC experiments, 30 µL RBCs were incubated in 0.3 mL total volume using PBS with 5mM glucose.

Calculations of DHA in media in moles accumulated in RBC in moles.

For fig3A: experimental volume of 0.5 mL has 2 µM DHA, or 1 nmole DHA available for transport at time zero. Packed RBC volume added is 50 µL. Internal RBC volume is 70% of packed RBC volume (Mendiratta et al., 1998) or 35 µL. RBC ascorbate values rise from ~ 32 µM to ~ 60 µM, increase of ~28 µM. For 35 µL volume, this is increase of 28 pmoles/µL X 35 µL = 0.98 nmoles, or ~ 1 nmole.

For supplemental fig3A,: experimental volume of 0.5 mL has 5 µM DHA, or 2.5 nmoles of DHA available for transport at time zero. Packed RBC volume added is 50 uL. Internal RBC volume is 70% of packed RBC volume (Mendiratta et al., 1998) or 35 µL. RBC ascorbate values rise from ~ 40 µM to ~ 110 µM, increase of ~ 70 µM. For 35 µL volume, this is increase of 70 pmoles/µL X 35 µL = 2.45 nmoles, or ~ 2.5 nmoles.

Supplementary Figure 4 A,B

Only ascorbate is found in RBCs

Human or mouse RBCs 40% were lysed with water at 37°C, 10 µM DHA was added, and samples obtained within 10 seconds for ascorbate measurement by HPLC. Samples are compared to ascorbate recovery from DHA with TCEP control (500 µM) (Li et al., 2012). Water lysis was used to remove substrate transport as a variable. In A left side, original data are shown including endogenous ascorbate. Symbols are: open bars, ascorbate in RBC lysate; grey bars, measured ascorbate in RBC lysate with 10 µM DHA added; dotted bars, predicted ascorbate in

RBC lysate with 10 μM DHA added. In A right side, data are shown with baselines subtracted. In B, data from a separate experiment as described in A are shown, with baseline subtracted.

Supplementary Figure 5

Inhibition of glucose analog uptake and DHA uptake by excess glucose analog

A. For study of glucose inhibition of DHA uptake, a requisite control was inhibition of radiolabelled [^{3}H]2-deoxyglucose uptake by excess cold glucose substrate (Cloherty et al., 1996; Carruthers and Naftalin, 2009; Carruthers et al., 2009). Human RBCs at 37^{0}C were incubated with 1.7 nM [^{3}H]2-deoxyglucose alone (○); 1.7 nM [^{3}H]2-deoxyglucose + 5 mM (Δ) or 25 mM glucose (□) without (left side) or with (right side) preincubation with the same concentration of glucose used for testing transport inhibition. Absence of inhibition, as observed, could be explained by confounding factors: metabolism of 2-deoxyglucose and/or glucose after transport; rapidity of glucose transport at 37°C, so that substrate was not in excess; lack of accounting for trans-activation; or these factors in combination(Stein, 1986) (Cloherty et al., 1996; Carruthers and Naftalin, 2009; Carruthers et al., 2009). Subsequent experimental conditions in the following figures were modified as follows: conduct of transport assays at 4°C; use of the non-metabolized substrate 3-O-MG; pre-loading RBCs with 3-O-MG to account for transactivation.

B. Time course for loading human RBCs with 5 (○) or 20 (□) mM 3-O-MG at 37°C. Human RBCs are fully preloaded by 20 minutes.

C,D. Effect of preloading human RBCs with varying 3-O-MG concentrations on subsequent uptake of 5 or 20 mM 3-O-MG. Human RBCs were incubated at 37°C for 20 minutes with 3-O-MG concentrations of 0 – 20 mM. Samples were centrifuged, supernatant removed, and pre-loaded RBCs kept on ice until used. Transport of 5 (○) and 20 (□) mM [^{3}H]3-O-MG as a

function of varying pre-loading concentrations was measured at 4°C for 1 minute, data displayed as calculated values (C) and cpm (D).

E. Inhibition of uptake of 10 µM [^{3}H] 3-O-MG into human RBCs over 1 minute at 4°C by increasing concentrations of unlabelled 3-O-MG as shown. RBCs were pre-loaded with 20 mM 3-O-MG for 20 minutes at 37°C (see B-D). Transport time of 1 minute was chosen because shorter transport times could underestimate 3-O-MG inhibition of DHA transport in subsequent experiments.

Supplementary Figure 6

Reduction of DHA by human RBC lysates (40%) at 4°C compared to 37°C

Reduction of 5 µM DHA was always measured at 10 seconds, with controls at 37°C and reduction of standard (DHA alone) with TCEP. The left panel indicates ascorbate data with background RBC ascorbate included. Open bars, ascorbate in RBC lysate; grey bars, measured ascorbate in RBC lysate with 5 µM DHA added at indicated temperature; dotted bars, predicted ascorbate in RBC lysate with 5 µM DHA added at indicated temperature. Right panel shows data with background subtracted and includes controls for TCEP reduction of DHA and reduction by lysate at 37°C.

Supplementary Figure 7

RBC ascorbate as a function of plasma ascorbate in subjects with and without hyperglycemia at the time samples were obtained

(■, —), subjects without hyperglycemia (glucose < 6.9 mM / 125 mg/dl); (◆, —), subjects with hyperglycemia (glucose > 6.9 mM/ 125 mg/dl). RBC ascorbate : 47.3 +/- 15 in subjects

without hyperglycemia vs. 31.1 ± 14 in subjects with hyperglycemia, $p < 0.01$ (t-test). Subjects without hyperglycemia $R = 0.93$; subjects with hyperglycemia $R = 0.98$. Subjects who had diabetes but who were euglycemic at the time of testing are indicated by ◇, and subjects with impaired glucose tolerance but who were euglycemic at the time of testing are indicated by ■. Definitions are from the American Diabetes Association(American Diabetes Association: Position Statement, 2014): normal: fasting glucose is < 100 mg/dl, hemoglobin A1C < 5.7; impaired glucose tolerance: fasting glucose 100-125, hemoglobin A1C 5.7-6.4; diabetes: fasting glucose >125, hemoglobin A1C 6.5 or higher.

Reference List

American Diabetes Association: Position Statement (2014). Diagnosis and Classification of Diabetes Mellitus. Diabetes Care *37*, S81-S90.

Carruthers,A., DeZutter,J., Ganguly,A., and Devaskar,S.U. (2009). Will the original glucose transporter isoform please stand up! Am. J. Physiol Endocrinol. Metab. *297*, E836-E848.

Carruthers,A. and Naftalin,R.J. (2009). Altered GLUT1 substrate selectivity in human erythropoiesis? Cell *137*, 200-201.

Cloherty,E.K., Heard,K.S., and Carruthers,A. (1996). Human erythrocyte sugar transport is incompatible with available carrier models. Biochemistry. *35*, 10411-10421.

Corpe,C.P., Eck,P., Wang,J., Al-Hasani,H., and Levine,M. (2013). Intestinal dehydroascorbic acid (DHA) transport mediated by the facilitative sugar transporters, GLUT2 and GLUT8. J. Biol. Chem. *288*, 9092-9101.

Freckmann,G., Schmid,C., Baumstark,A., Pleus,S., Link,M., and Haug,C. (2012). System accuracy evaluation of 43 blood glucose monitoring systems for self-monitoring of blood glucose according to DIN EN ISO 15197. J. Diabetes Sci. Technol. *6*, 1060-1075.

Li,H., Tu,H., Wang,Y., and Levine,M. (2012). Vitamin C in mouse and human red blood cells: an HPLC assay. Anal. Biochem. *426*, 109-117.

Maeda,N., Hagihara,H., Nakata,Y., Hiller,S., Wilder,J., and Reddick,R. (2000). Aortic wall damage in mice unable to synthesize ascorbic acid. Proc. Natl. Acad. Sci. U. S. A *97*, 841-846.

Mendiratta,S., Qu,Z.C., and May,J.M. (1998). Erythrocyte ascorbate recycling: antioxidant effects in blood. Free Radic. Biol. Med *24*, 789-797.

Moitra,J., Mason,M.M., Olive,M., Krylov,D., Gavrilova,O., Marcus-Samuels,B., Feigenbaum,L., Lee,E., Aoyama,T., Eckhaus,M., Reitman,M.L., and Vinson,C. (1998). Life without white fat: a transgenic mouse. Genes Dev. *12*, 3168-3181.

Parpart, A.K., Lorenz, P.B., Parpart,E.R., Gregg,J.R., and Chase,A.M. (1947). The osmotic resistance (fragility) of human red cells. J. Clin. Invest *26*, 636-640.

Sacks,D.B. (2008). Carbohydrates. In Tietze Fundamentals of Clinical Chemistry, B.A.Burtis, E.R.Ashwood, D.E.Bruns, and B.G.Sawyer, eds. (St. Louis: Saunders Elsevier), pp. 373-401.

Stein,W.D. (1986). Facilitated Diffusion: the simple carrier. In Transport and Diffusion Across Cell Membranes, W.D.Stein, ed. (London, U.K.: Academic Press), pp. 231-362.

Suppl Fig 1A

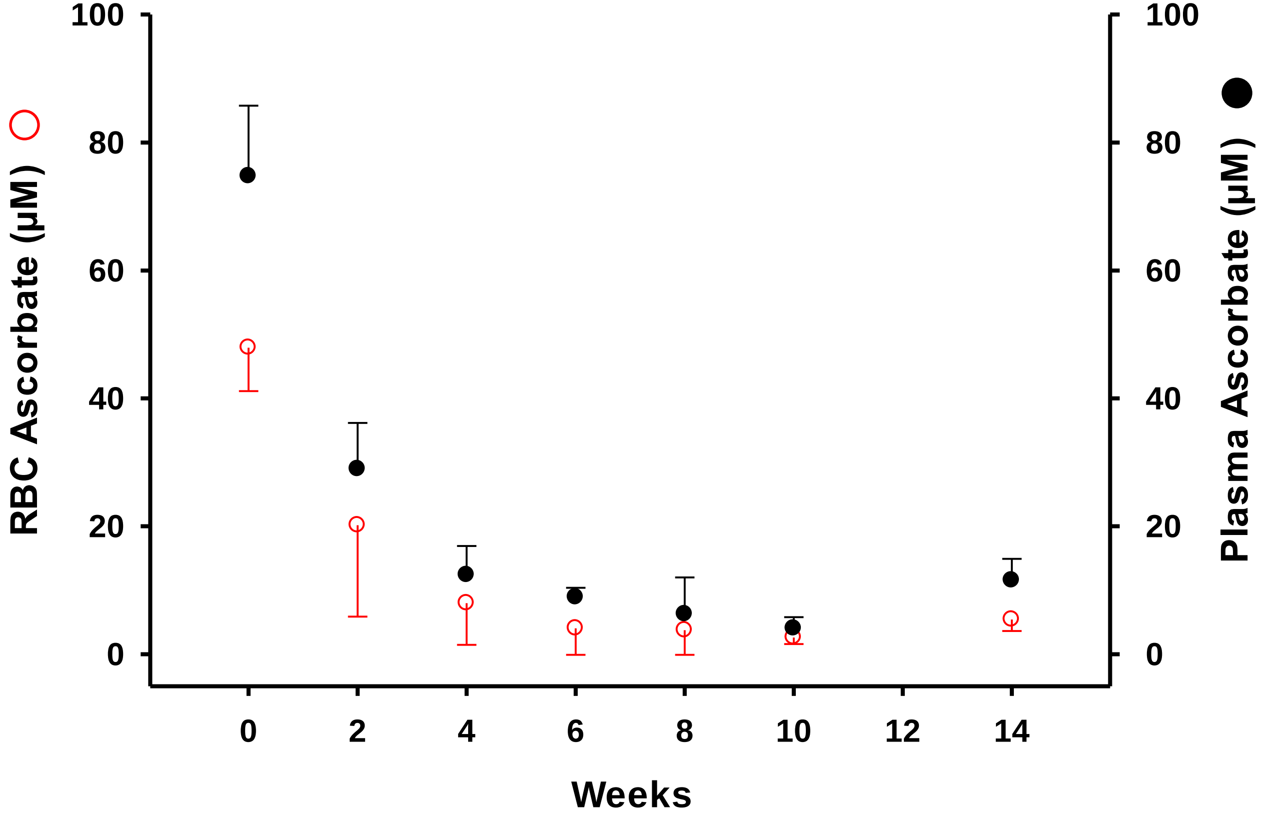

RBC Ascorbate (µM)
Plasma Ascorbate (µM)
Weeks
100
80
60
40
20
0
0
2
4
6
8
10
12
14

Suppl Fig 1B

Gulo +AA | Gulo -AA

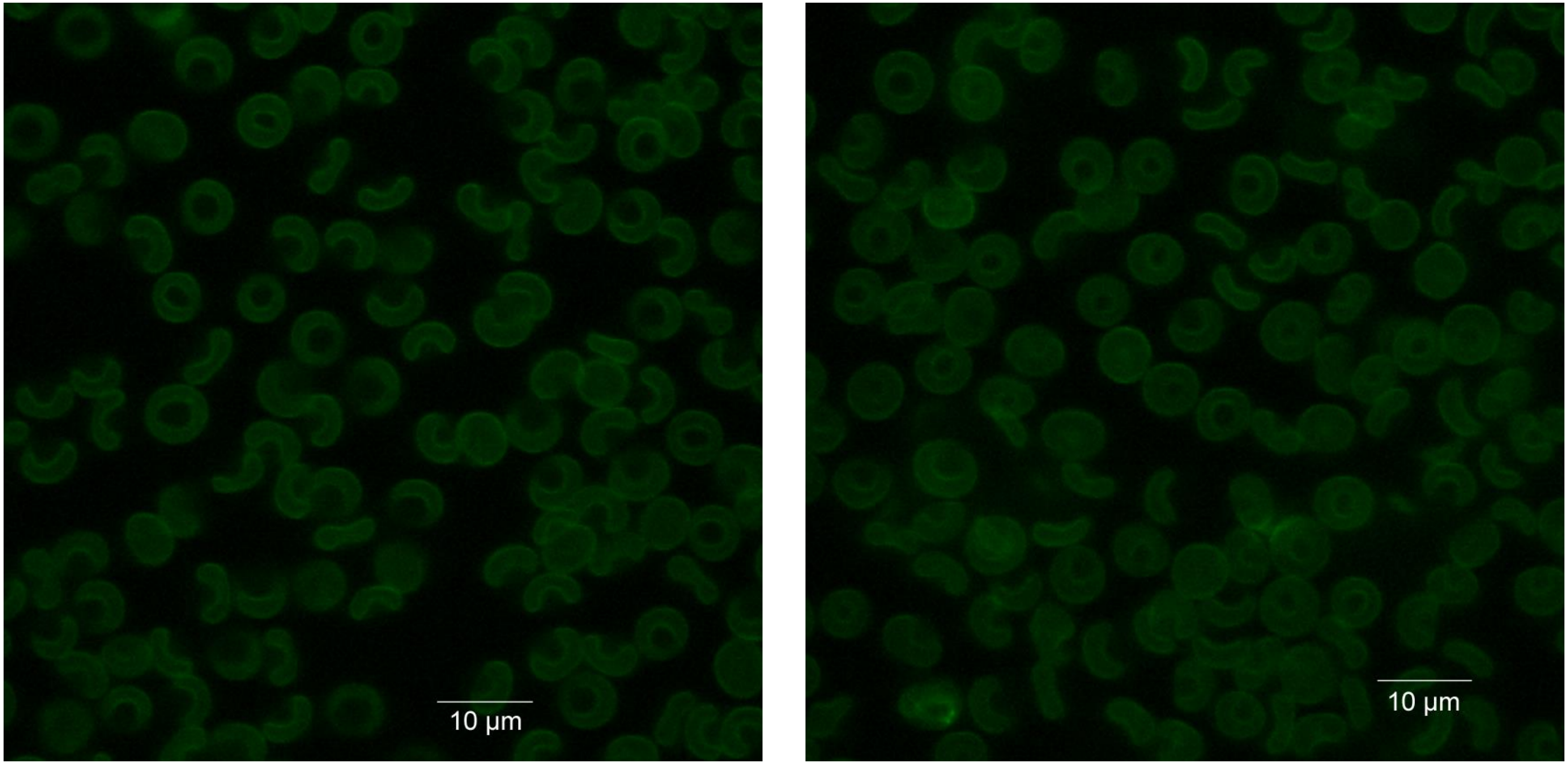

Suppl Fig 1C

Gulo +AA

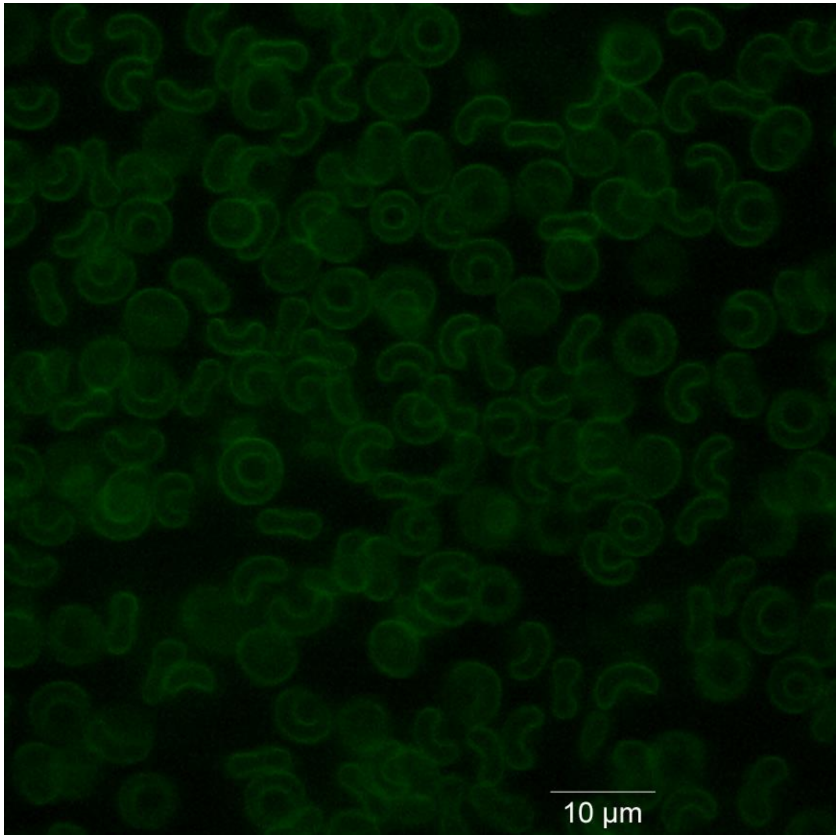

Gulo –AA recovery

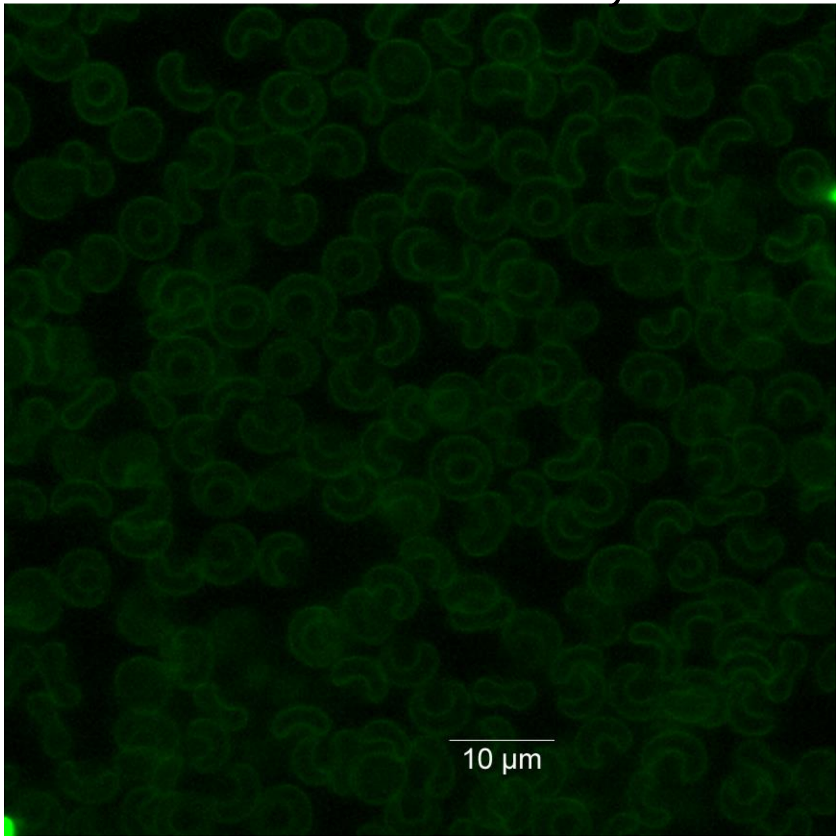

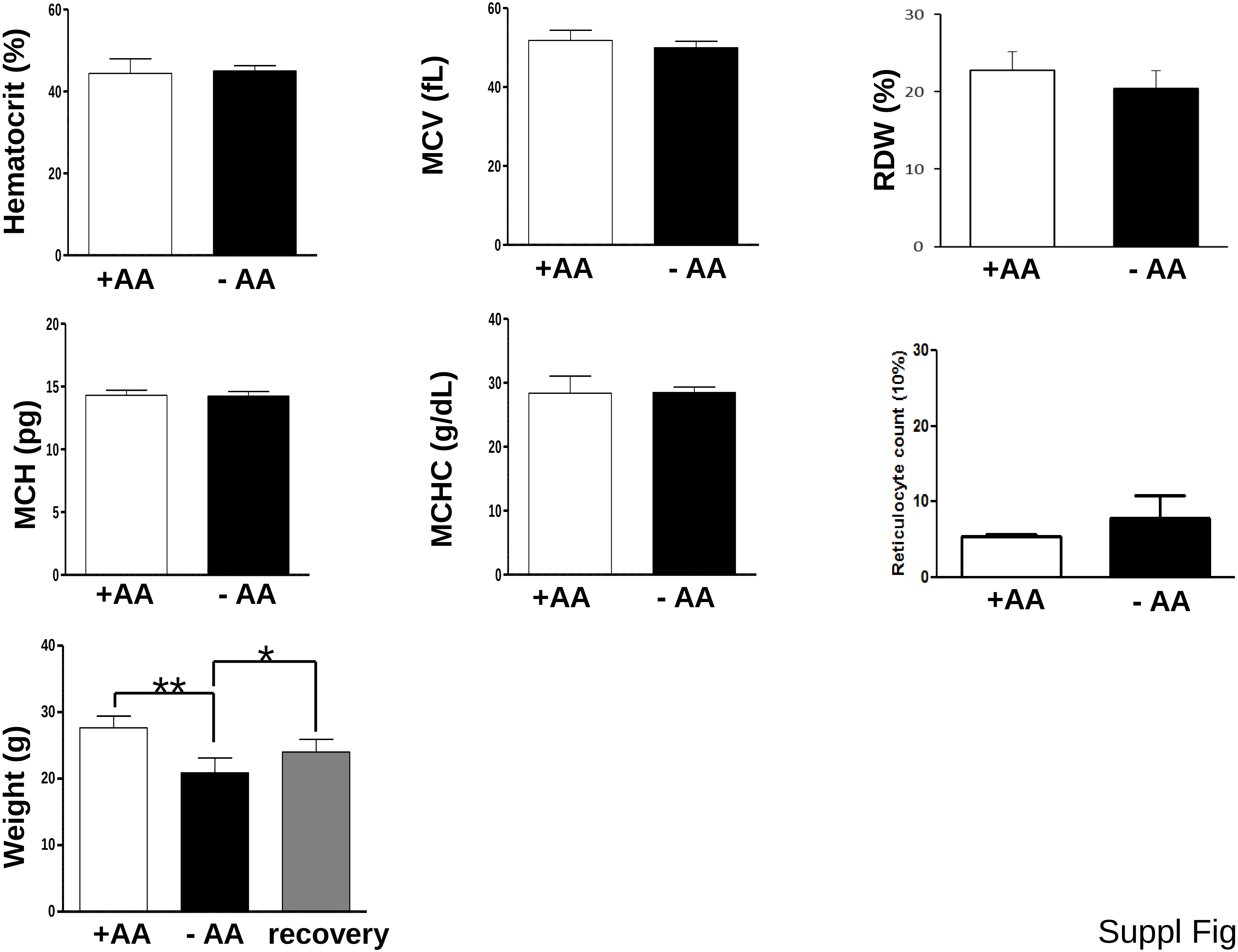
Hematocrit (%)
60
40
20
0
+AA
- AA
MCV (fL)
60
40
20
0
+AA
- AA
RDW (%)
30
20
10
0
+AA
- AA
MCH (pg)
20
15
10
5
0
+AA
- AA
MCHC (g/dL)
40
30
20
10
0
+AA
- AA
Reticulocyte count (10%)
30
20
10
0
+AA
- AA
Weight (g)
40
30
20
10
0
**
*
+AA
- AA
recovery

Suppl Fig 1D

Suppl Fig 1E

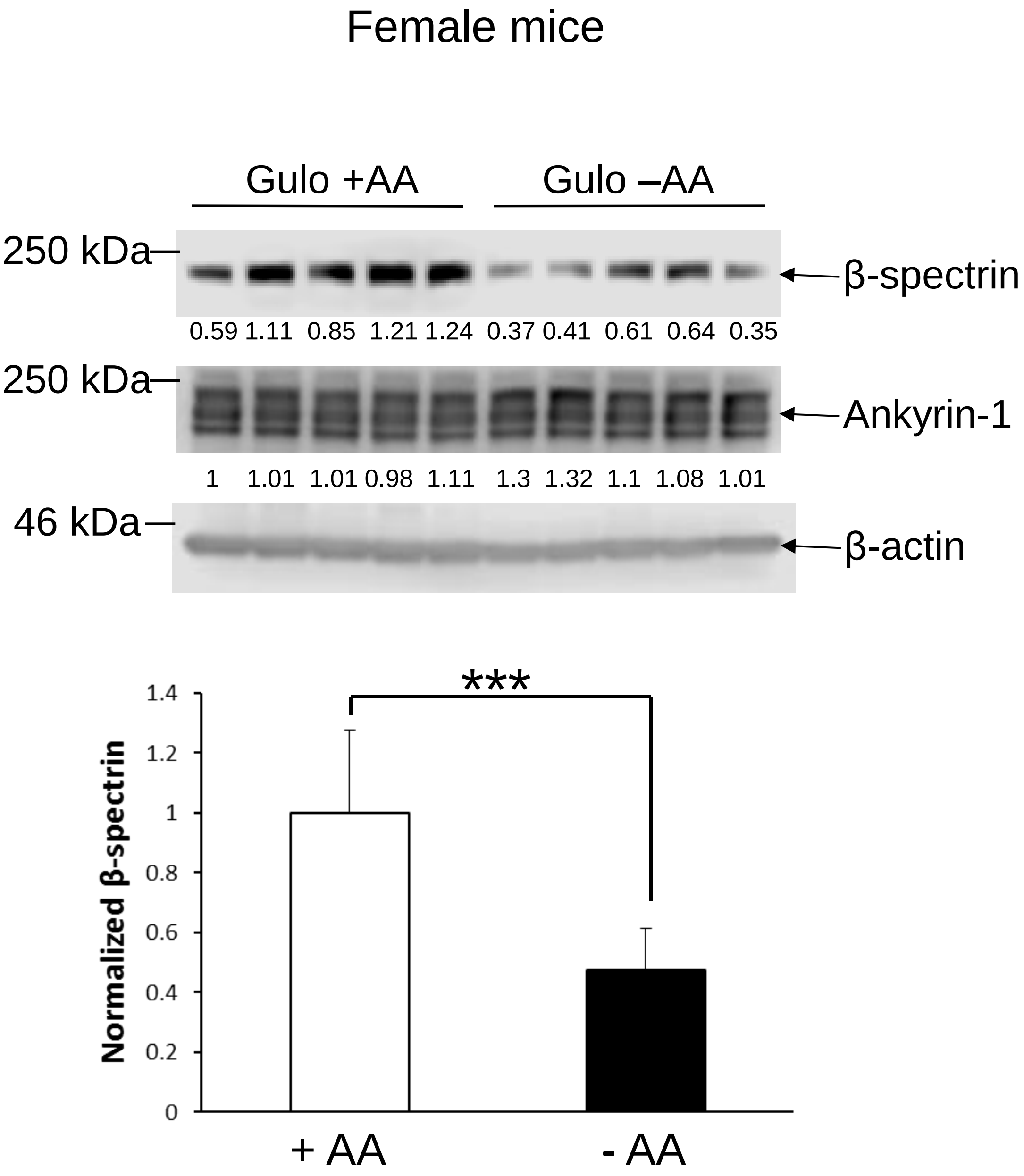
Female mice
Gulo +AA
Gulo –AA
250 kDa
β-spectrin
0.59 1.11 0.85 1.21 1.24 0.37 0.41 0.61 0.64 0.35
250 kDa
Ankyrin-1
1 1.01 1.01 0.98 1.11 1.3 1.32 1.1 1.08 1.01
46 kDa
β-actin
***
Normalized β-spectrin
1.4
1.2
1
0.8
0.6
0.4
0.2
0
+ AA
- AA

Suppl Fig 1F

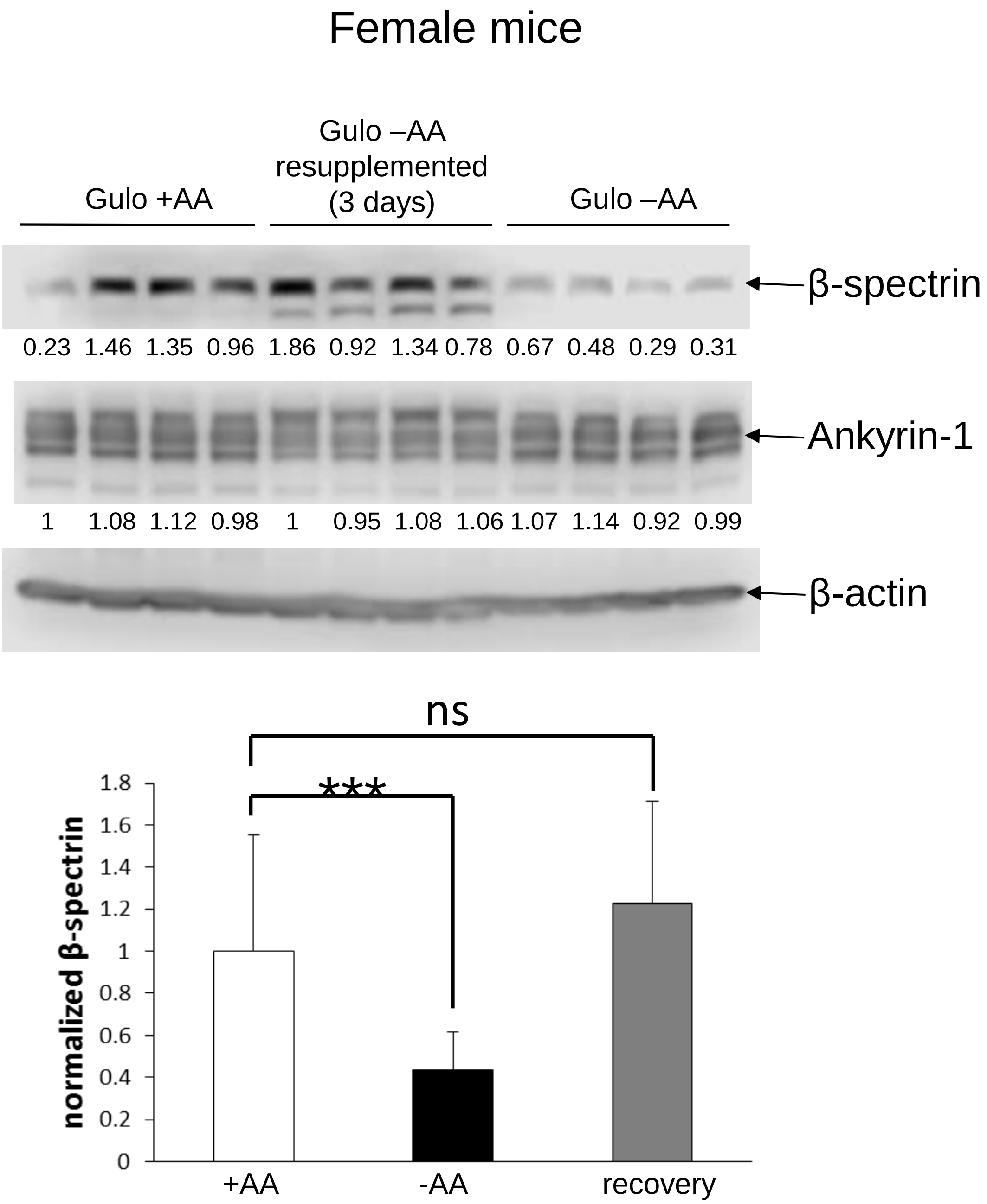

Female mice
Gulo +AA
Gulo –AA resupplemented (3 days)
Gulo –AA
β-spectrin
0.23 1.46 1.35 0.96 1.86 0.92 1.34 0.78 0.67 0.48 0.29 0.31
Ankyrin-1
1 1.08 1.12 0.98 1 0.95 1.08 1.06 1.07 1.14 0.92 0.99
β-actin
ns
***
normalized β-spectrin
1.8
1.6
1.4
1.2
1
0.8
0.6
0.4
0.2
0
+AA
-AA
recovery

Suppl Fig 1G

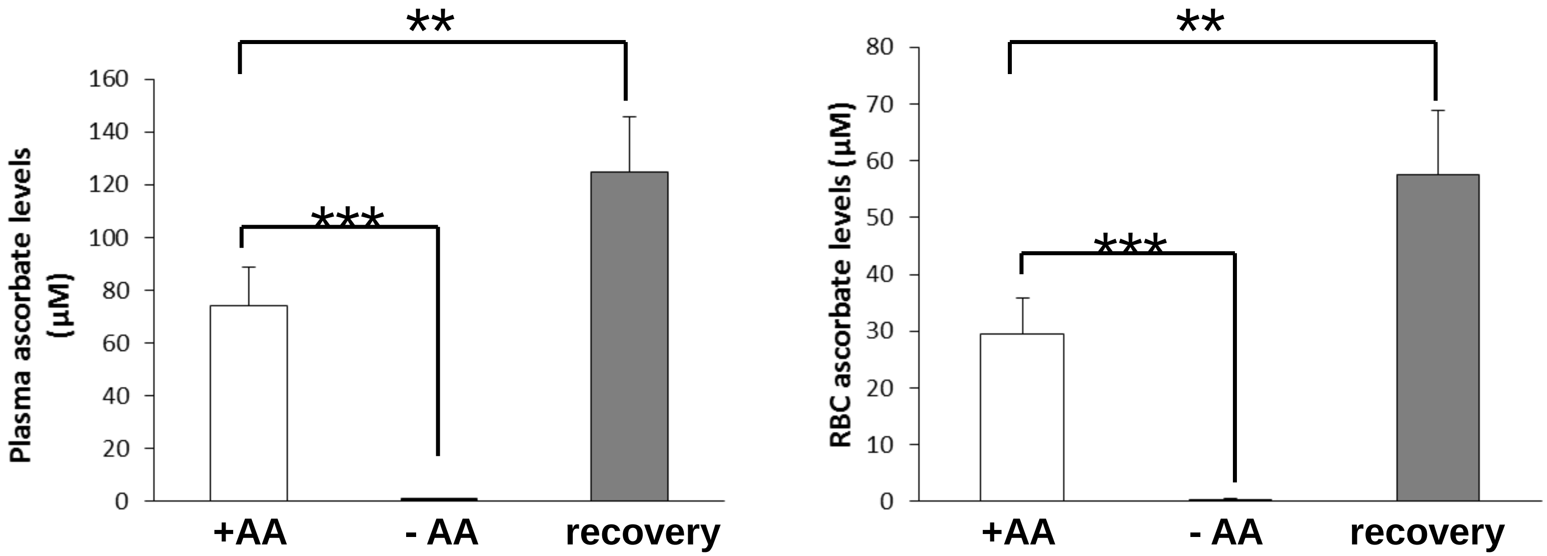

**
***
Plasma ascorbate levels (µM)
160
140
120
100
80
60
40
20
0
+AA
- AA
recovery
**
***
RBC ascorbate levels (µM)
80
70
60
50
40
30
20
10
0
+AA
- AA
recovery

Suppl Fig 1H

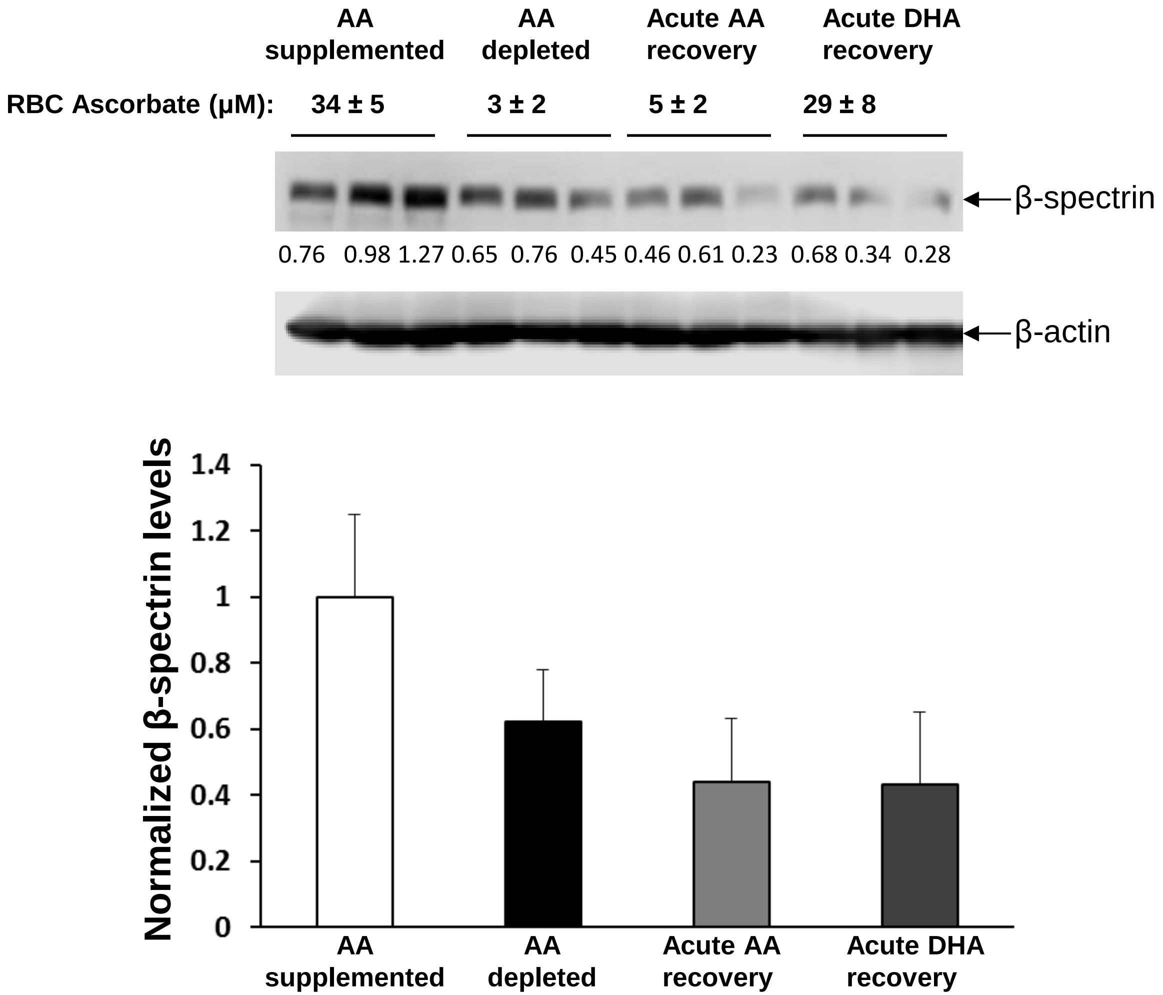
AA supplemented
AA depleted
Acute AA recovery
Acute DHA recovery
RBC Ascorbate (µM):
34 ± 5
3 ± 2
5 ± 2
29 ± 8
β-spectrin
0.76 0.98 1.27 0.65 0.76 0.45 0.46 0.61 0.23 0.68 0.34 0.28
β-actin
Normalized β-spectrin levels
1.4
1.2
1
0.8
0.6
0.4
0.2
0
AA supplemented
AA depleted
Acute AA recovery
Acute DHA recovery

Suppl Fig 2

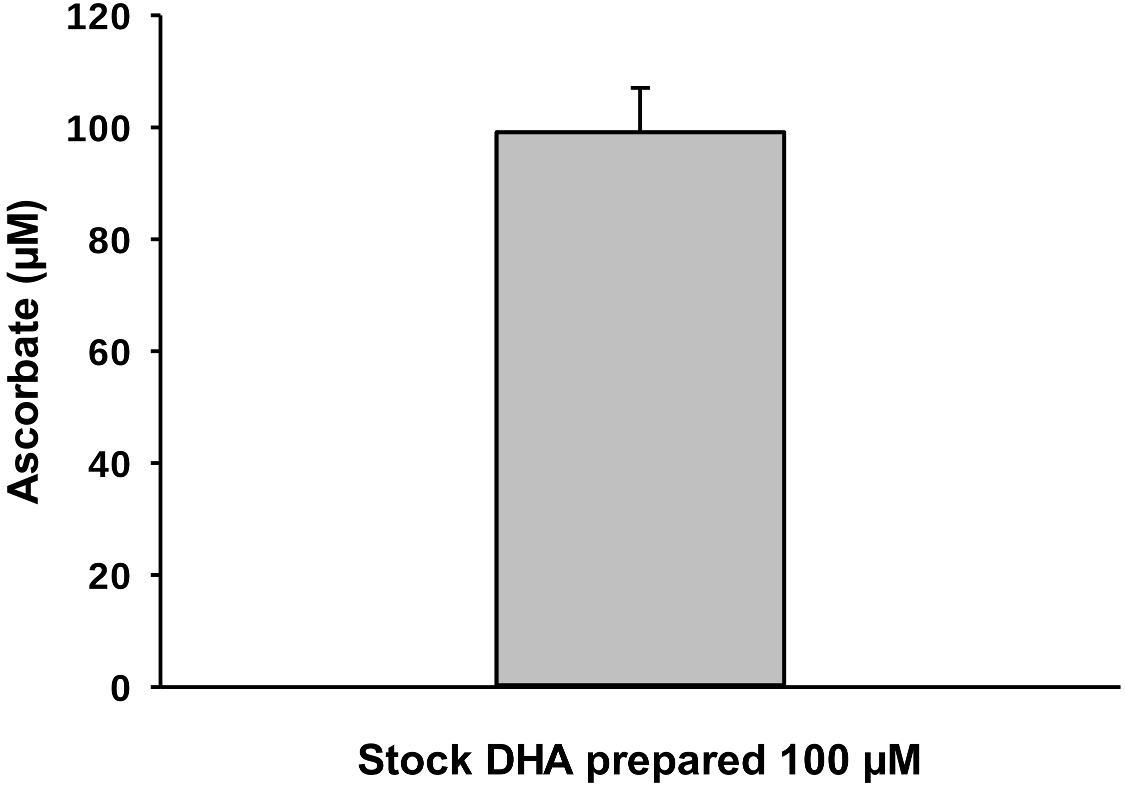

Suppl Fig 3A

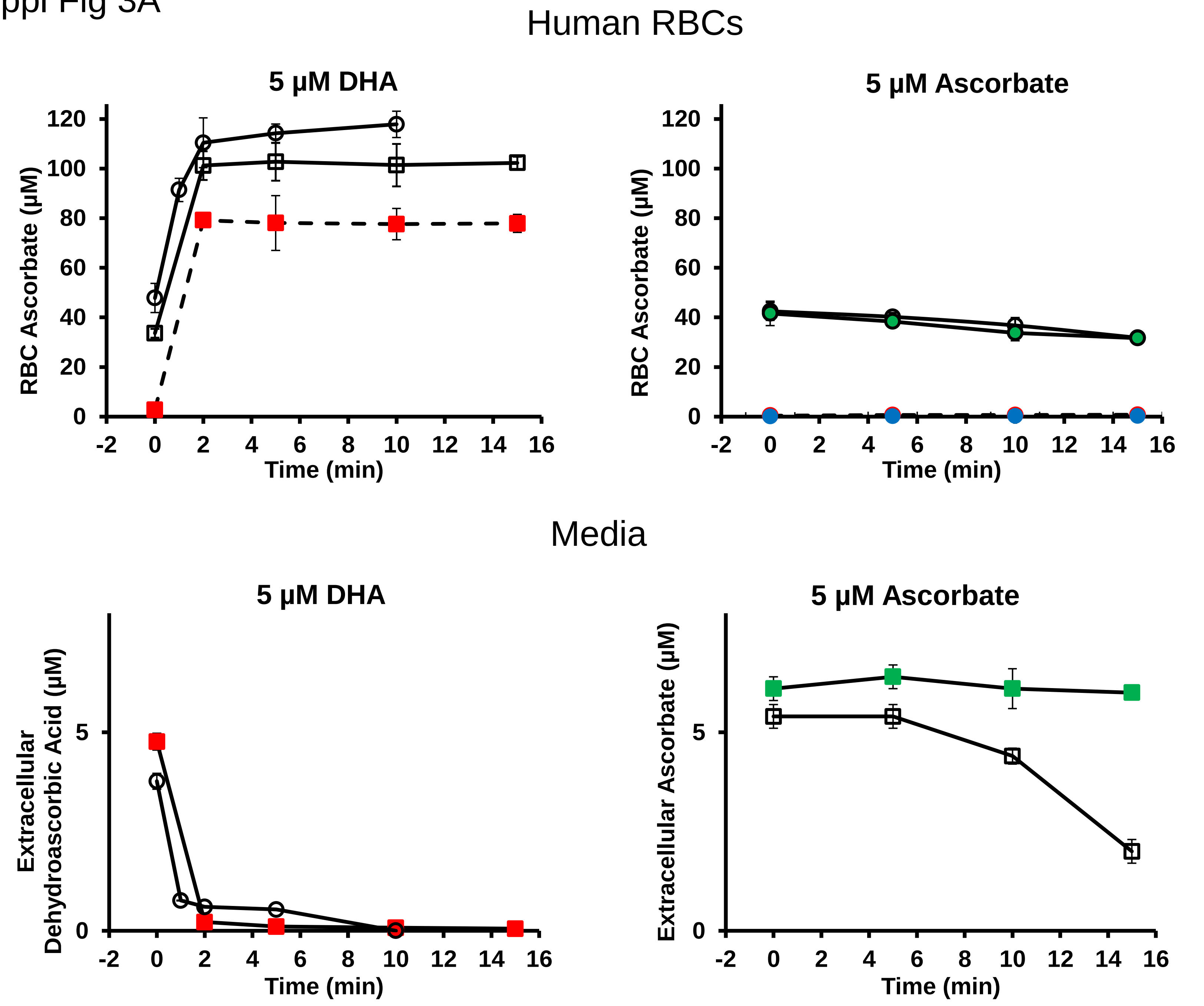

Human RBCs
5 µM DHA
RBC Ascorbate (µM)
120
100
80
60
40
20
0
-2
0
2
4
6
8
10
12
14
16
Time (min)
5 µM Ascorbate
RBC Ascorbate (µM)
Time (min)
Media
5 µM DHA
Extracellular
Dehydroascorbic Acid (µM)
5
0
Time (min)
5 µM Ascorbate
Extracellular Ascorbate (µM)
Time (min)

Suppl Fig 3B

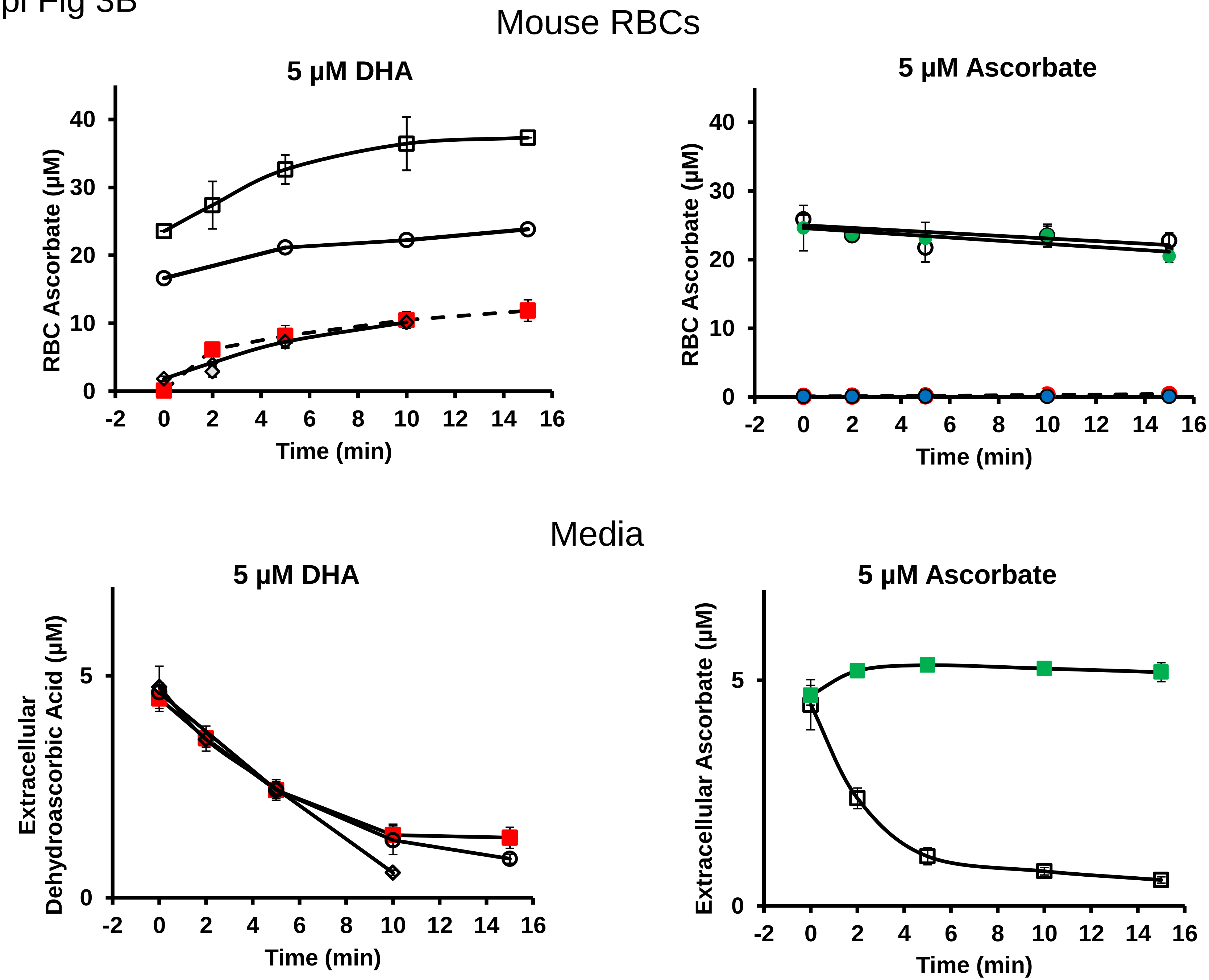
Mouse RBCs
5 µM DHA
RBC Ascorbate (µM)
40
30
20
10
0
-2 0 2 4 6 8 10 12 14 16
Time (min)
5 µM Ascorbate
RBC Ascorbate (µM)
40
30
20
10
0
-2 0 2 4 6 8 10 12 14 16
Time (min)
Media
5 µM DHA
Extracellular
Dehydroascorbic Acid (µM)
5
0
-2 0 2 4 6 8 10 12 14 16
Time (min)
5 µM Ascorbate
Extracellular Ascorbate (µM)
5
0
-2 0 2 4 6 8 10 12 14 16
Time (min)

Suppl Fig 4A

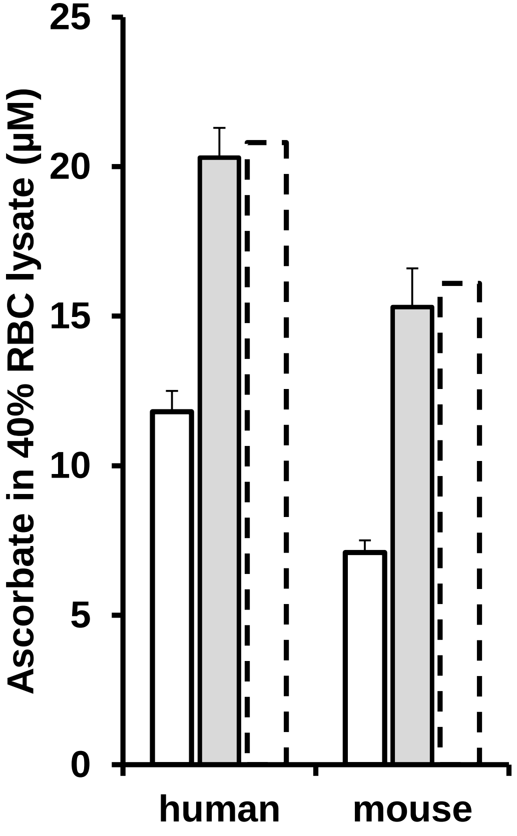

25
20
15
10
5
0
Ascorbate in 40% RBC lysate (µM)
human
mouse

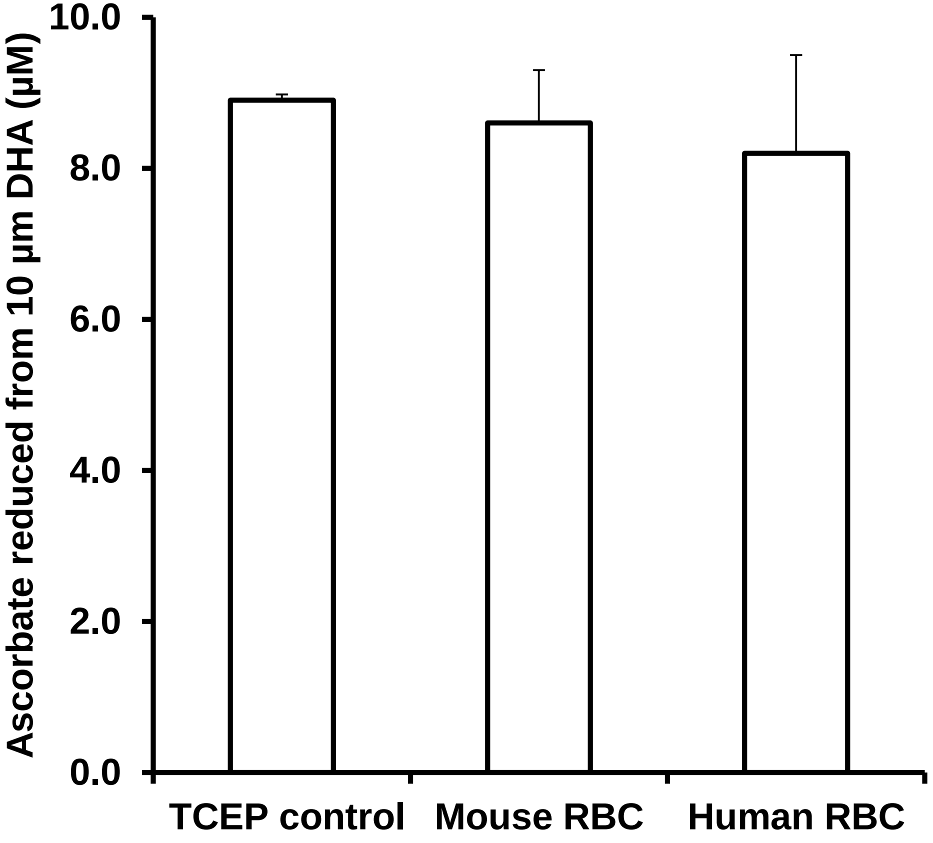

10.0
8.0
6.0
4.0
2.0
0.0
Ascorbate reduced from 10 µm DHA (µM)
TCEP control
Mouse RBC
Human RBC

Suppl Fig 4B

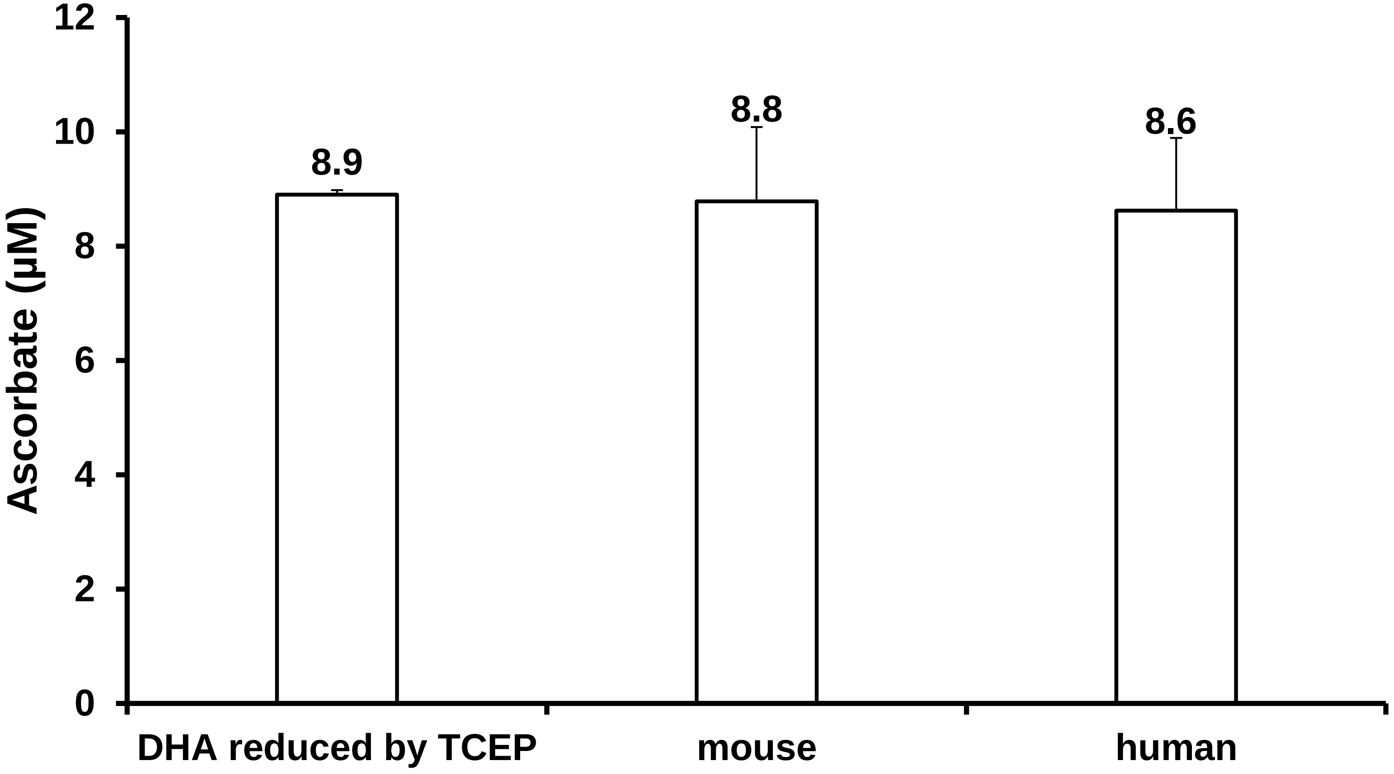
Ascorbate (µM)
12
10
8
6
4
2
0
8.9
8.8
8.6
DHA reduced by TCEP
mouse
human

Suppl Fig 5A

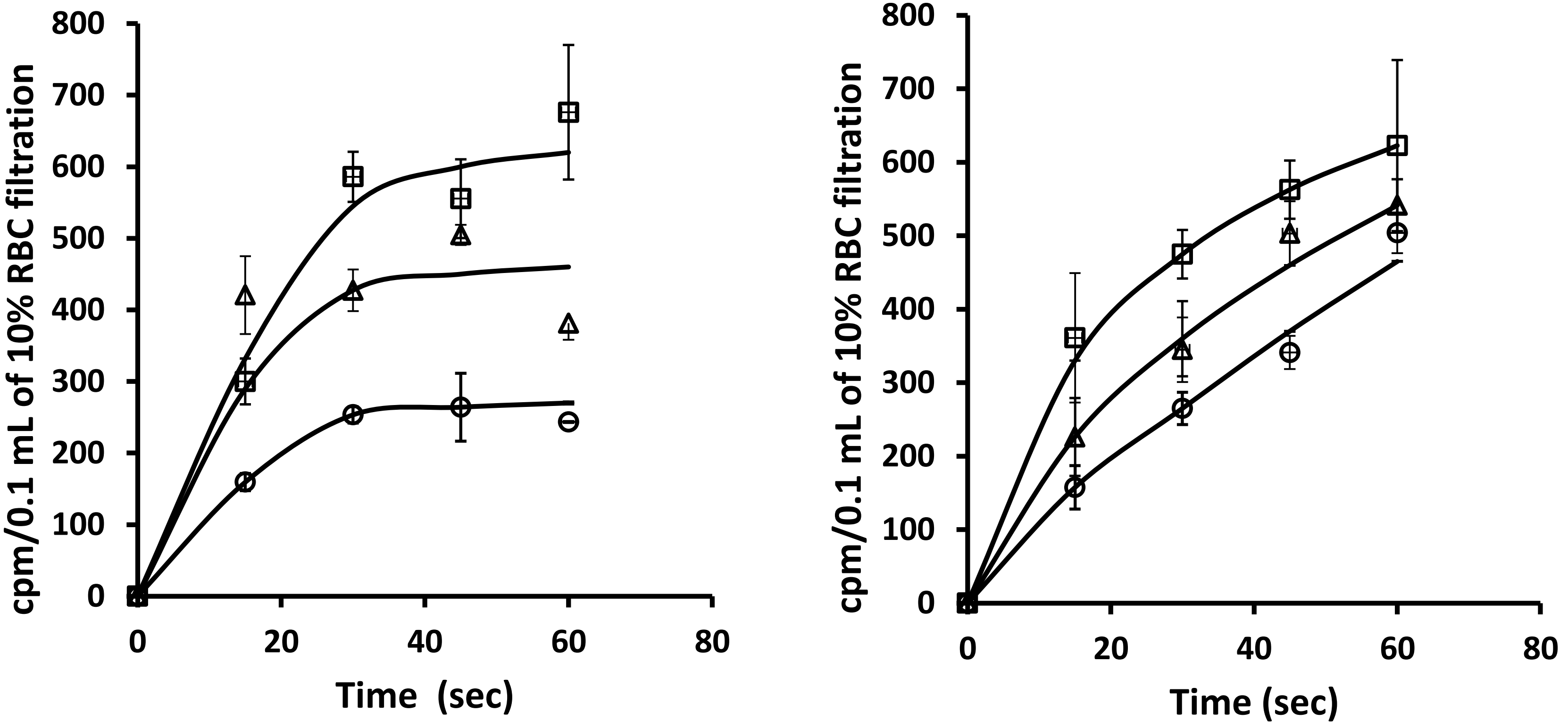

800
700
600
500
400
300
200
100
0
cpm/0.1 mL of 10% RBC filtration
0
20
40
60
80
Time (sec)
800
700
600
500
400
300
200
100
0
cpm/0.1 mL of 10% RBC filtration
0
20
40
60
80
Time (sec)

Suppl Fig 5B

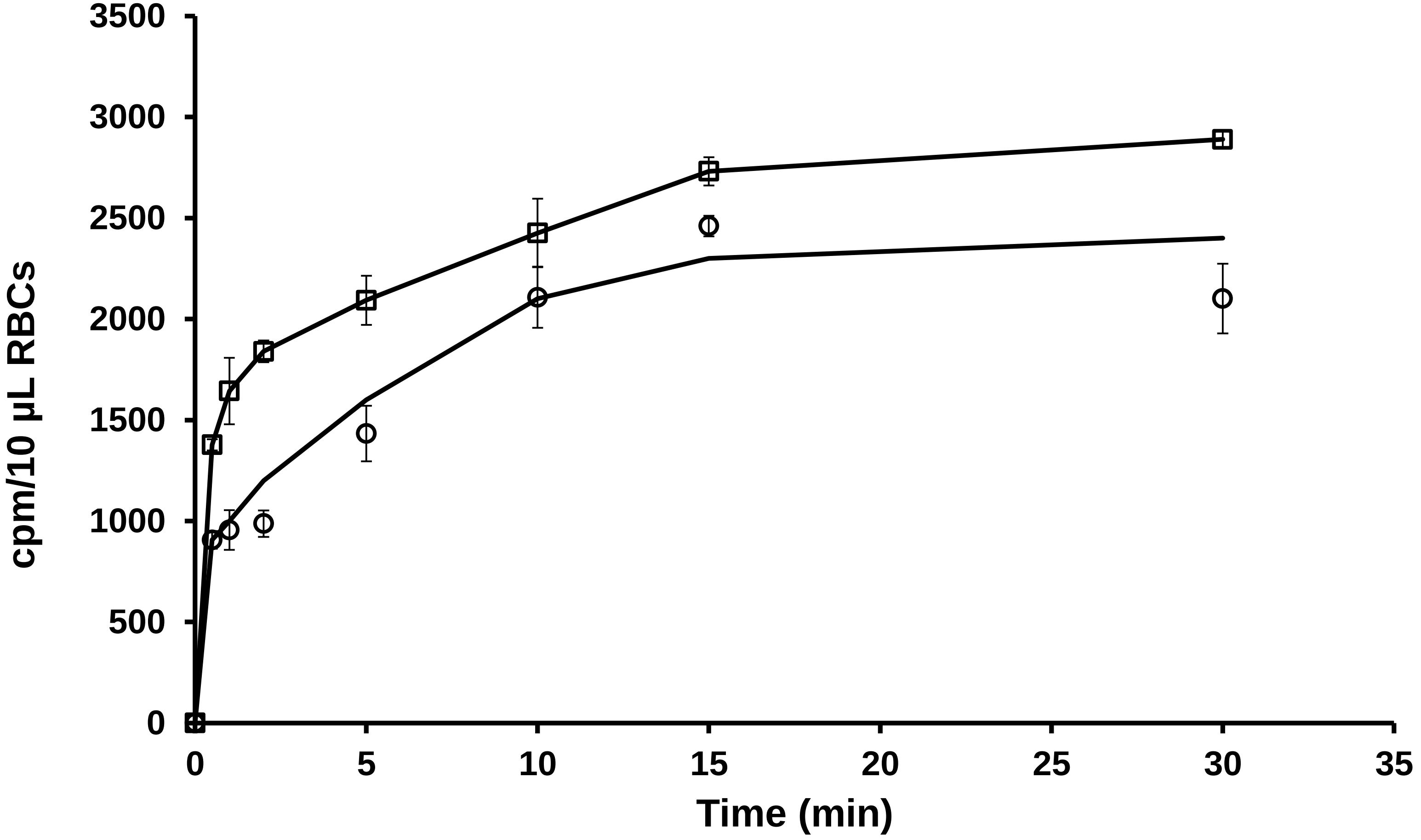
3500
3000
2500
2000
1500
1000
500
0
cpm/10 µL RBCs
0
5
10
15
20
25
30
35
Time (min)

Suppl Fig 5C

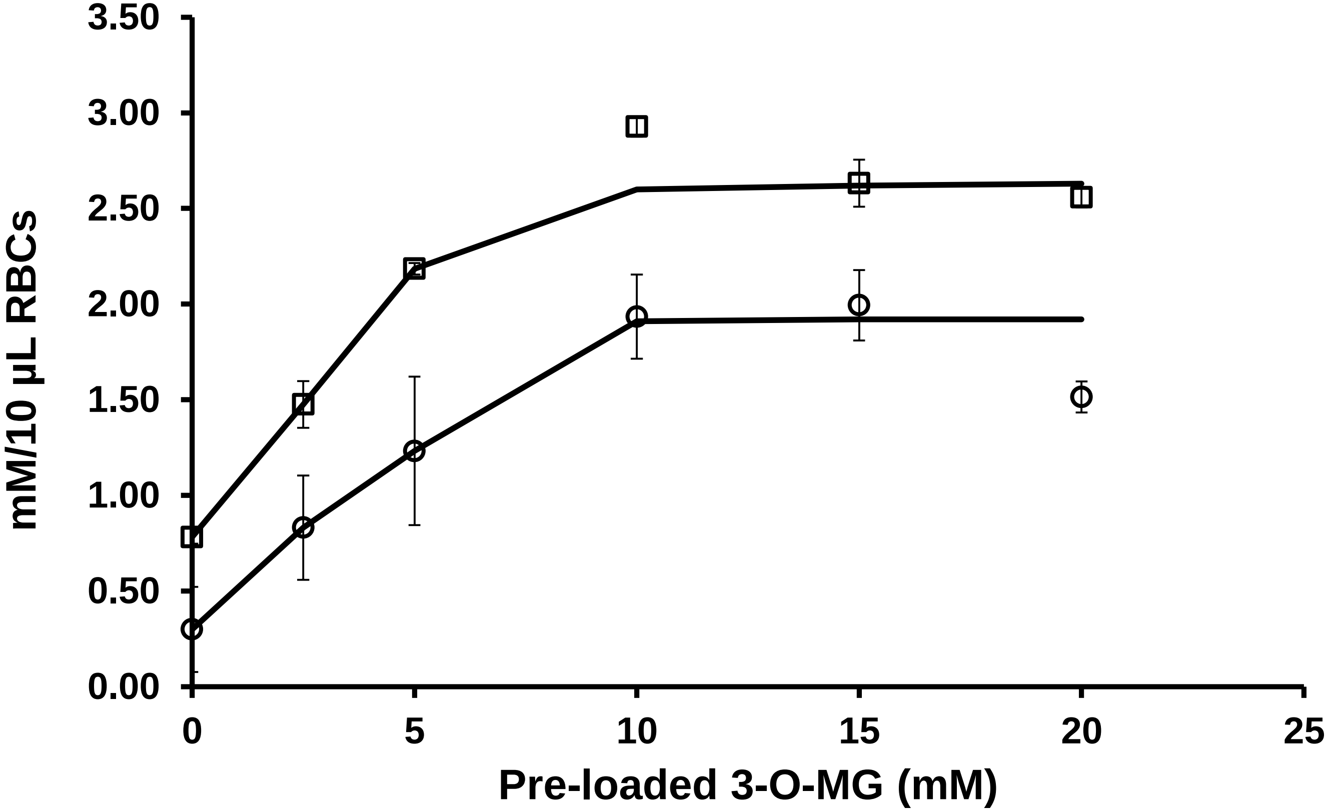

3.50
3.00
2.50
2.00
1.50
1.00
0.50
0.00
mM/10 µL RBCs
0
5
10
15
20
25
Pre-loaded 3-O-MG (mM)

Suppl Fig 5D

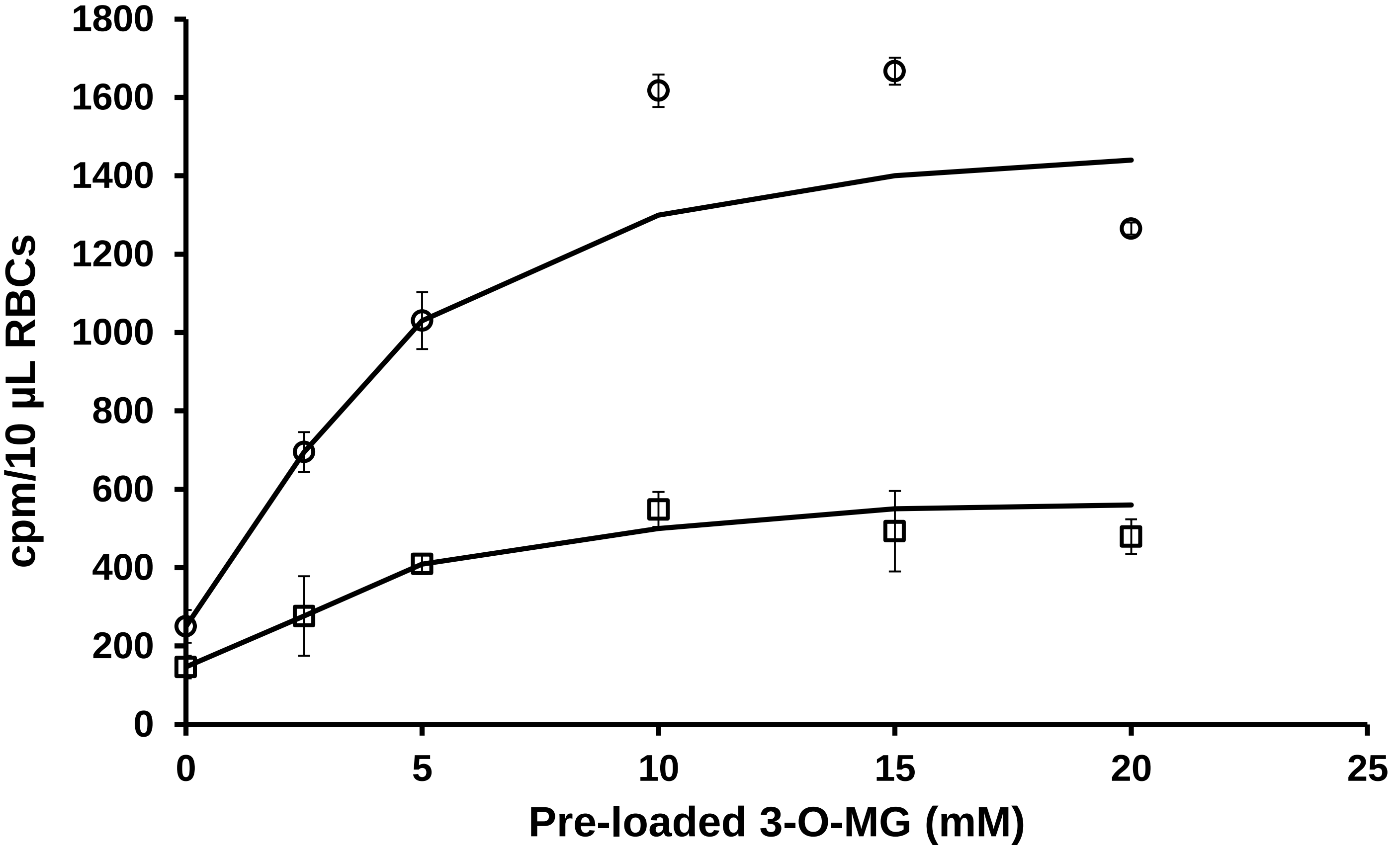

1800
1600
1400
1200
1000
800
600
400
200
0
cpm/10 µL RBCs
0
5
10
15
20
25
Pre-loaded 3-O-MG (mM)

Suppl Fig 5E

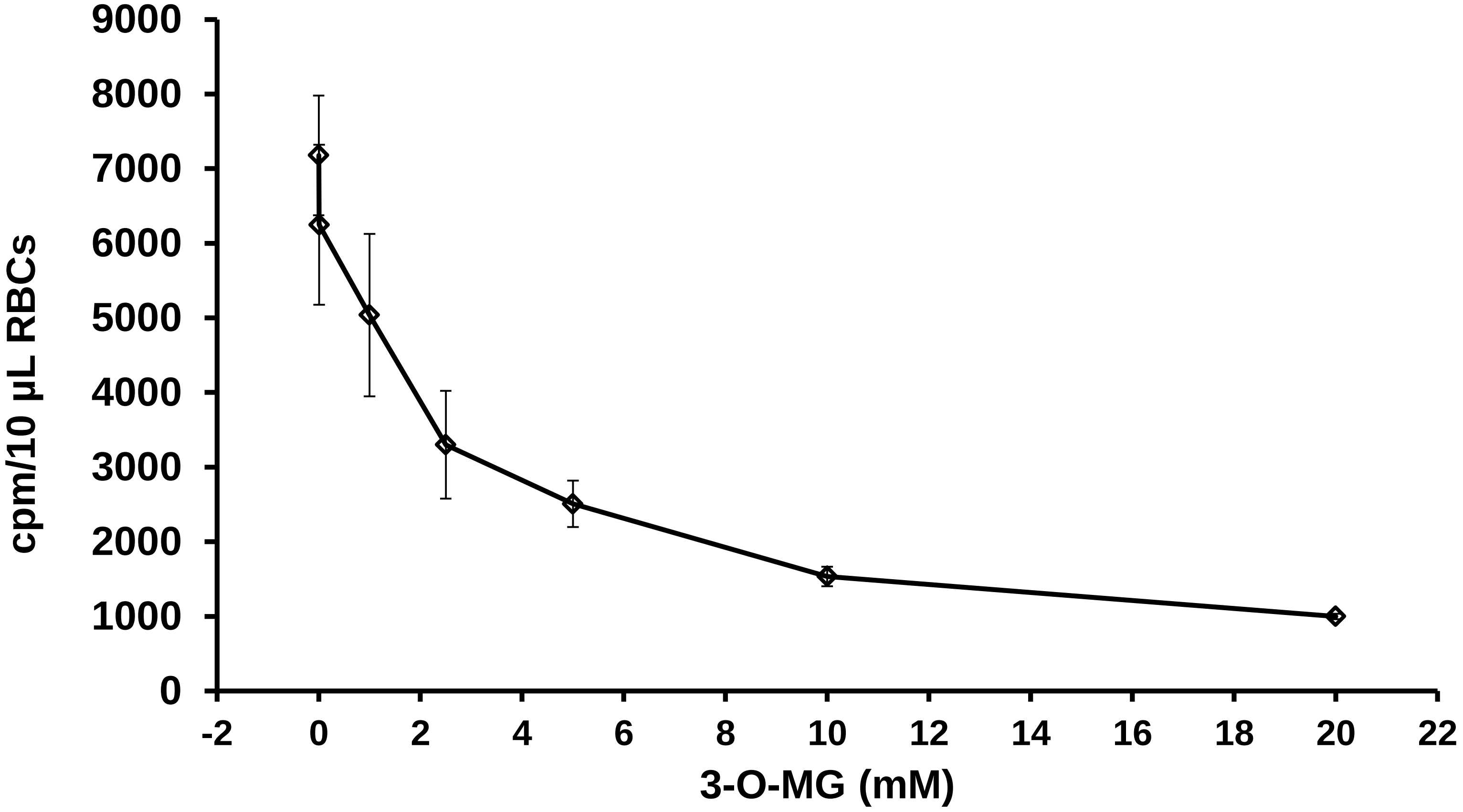

9000
8000
7000
6000
5000
4000
3000
2000
1000
0
cpm/10 µL RBCs
-2
0
2
4
6
8
10
12
14
16
18
20
22
3-O-MG (mM)

Suppl Fig 6

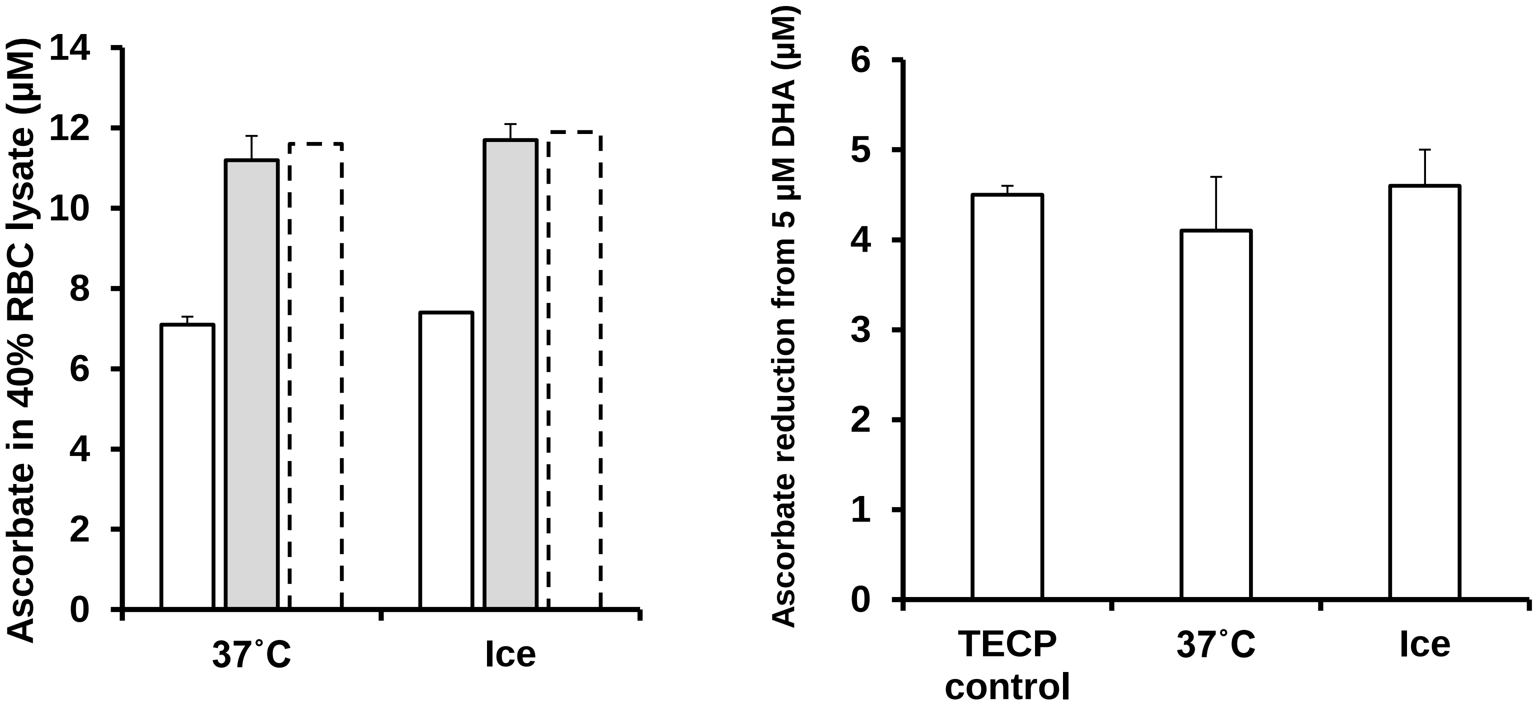

Ascorbate in 40% RBC lysate (µM)
14
12
10
8
6
4
2
0
37˚C
Ice
Ascorbate reduction from 5 µM DHA (µM)
6
5
4
3
2
1
0
TECP control
37˚C
Ice

Suppl Fig 7

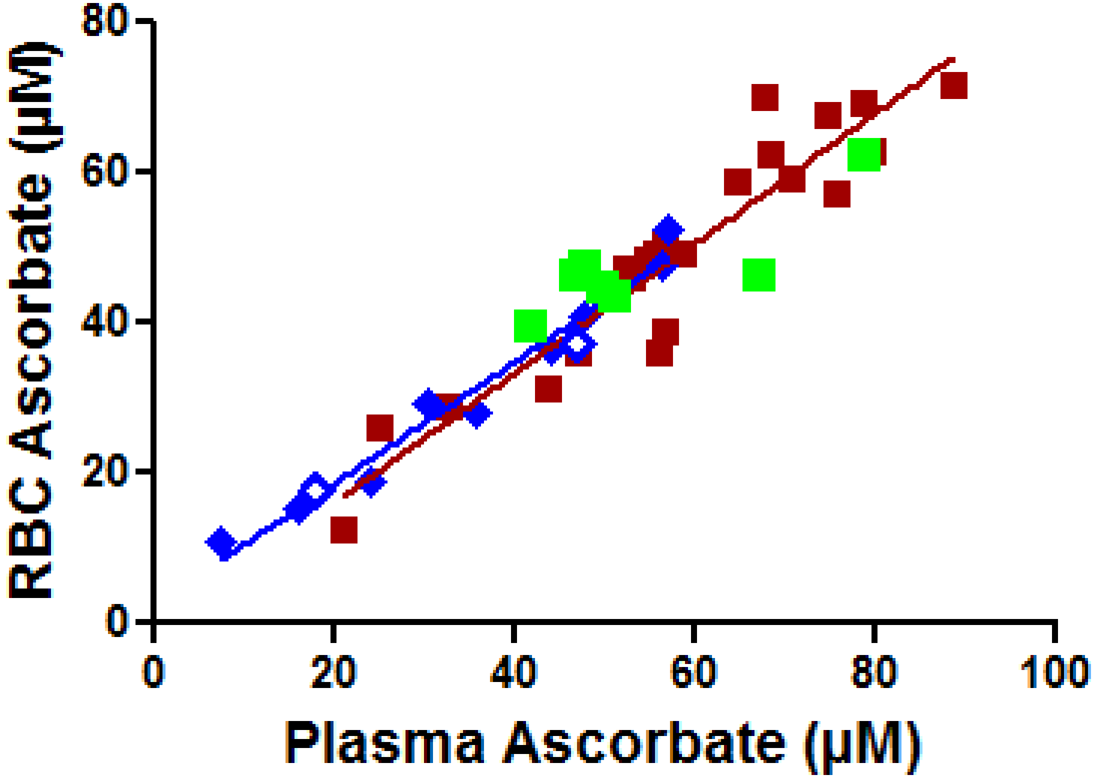

RBC Ascorbate (μM)
80
60
40
20
0
0
20
40
60
80
100
Plasma Ascorbate (μM)
